# GIPC2 regulation of the PKM2/SREBP1 signaling axis controls adipogenic differentiation of mesenchymal stem cells

**DOI:** 10.1038/s41419-025-08088-9

**Published:** 2026-01-07

**Authors:** Jiayi Wang, Chengqi Xin, Zhaokai Sun, Mengke Zhao, Yaoyao Zan, Zhongyue Lv, Shuaiyu Zhu, Jing Liu, Liang Wang

**Affiliations:** National Joint Engineering Laboratory, The First Affiliated Hospital of Dalian Medical University, Dalian City, Liaoning Province P.R. China

**Keywords:** Cell biology, Stem cells

## Abstract

Mesenchymal stem cell (MSC) differentiation is a cornerstone of regenerative medicine with a wide range of applications in tissue engineering and translational therapies. However, the molecular mechanisms underlying MSC differentiation remain incompletely understood, preventing the full leveraging of their therapeutic potential. Central to these complex molecular networks are dynamic protein–protein interactions, with scaffolding proteins serving as master coordinators. GAIP-interacting protein C-terminus 2 (GIPC2) functions as an adaptor protein involved in mediating such interactions and may influence MSC fate by regulating differentiation-related signaling pathways. In this study, we identified GIPC2 as a novel regulator of adipogenic differentiation in human umbilical cord-derived MSCs (UC-MSCs). Mechanistically, GIPC2 interacts directly with pyruvate kinase M2 (PKM2) via its PDZ domain, promoting PKM2 nuclear translocation. In the nucleus, PKM2 facilitates the activation of sterol regulatory element-binding protein 1 (SREBP1), a transcription factor essential for lipid biosynthesis and adipocyte maturation. Our findings show that GIPC2 drives MSC adipogenic differentiation by orchestrating the PKM2–SREBP1 signaling axis. This study reveals a previously unrecognized regulatory mechanism, highlighting the pivotal role of GIPC2 at the intersection of metabolic regulation and transcriptional control. These insights not only deepen our understanding of MSC differentiation but also open new avenues for enhancing MSC-based therapeutic strategies.

## Introduction

GAIP-interacting protein C-terminus 2 (GIPC2), a scaffolding protein of the GIPC family, plays a crucial role in various cellular processes, including signal transduction, cytoskeletal organization, and cellular homeostasis. Structurally, GIPC2 contains conserved PDZ, GH1, and GH2 domains, enabling interactions with a wide range of signaling molecules and cellular pathways [1]. These features position GIPC2 as an important regulator of cellular plasticity and fate. GIPC2 has been predominantly studied in the context of tumor biology, where it regulates vital processes such as epithelial–mesenchymal transition (EMT), metabolic reprogramming, and cell survival [2]. For instance, GIPC2 downregulation is linked to promoted EMT, increased migratory capacity, and resistance to apoptosis in gastric cancer cells. In contrast, its overexpression inhibits tumor growth and metastasis by modulating multiple signaling cascades [3–5]. Furthermore, many of the processes influenced by GIPC2 in cancer—such as plasticity and metabolic adaptation—are also crucial for stem cell behavior, particularly in self-renewal, differentiation, and lineage commitment [6, 7]. These overlapping features suggest that GIPC2 may also exert regulatory effects in other cellular contexts, including mesenchymal stem cells (MSCs), by modulating similar molecular mechanisms.

MSCs are multipotent stem cells capable of differentiating into adipocytes, osteoblasts, and chondrocytes, making them valuable tools in regenerative medicine and cell-based therapies [8]. This differentiation potential relies on tightly regulated molecular mechanisms that govern MSC fate decisions and determine their therapeutic efficacy [9]. Among the various lineage pathways, adipogenic differentiation is especially significant due to its relevance in soft tissue regeneration, reconstructive fat grafting, and the treatment of metabolic disorders such as lipodystrophy [10]. Furthermore, adipogenesis also plays a fundamental role in the physiological maintenance of tissue homeostasis and repair. Dysregulation of this process has been implicated in several pathological conditions, including obesity, lipodystrophy, and age-related fat redistribution, underscoring the need to better understand its regulation [11–13]. Despite advances in stem cell research, major gaps remain in understanding the molecular networks that control MSC adipogenic differentiation. Addressing these gaps is essential for optimizing MSC-based therapeutic strategies and deepening our understanding of how transcriptional and metabolic signals coordinate to direct stem cell fate [14, 15].

The adipogenic differentiation of MSCs is orchestrated by complex transcriptional networks. A key regulator in this regulatory cascade is sterol regulatory element-binding protein 1 (SREBP1), a transcription factor that integrates lipid metabolic cues with adipocyte development [16–18]. SREBP1 governs the expression of genes involved in fatty acid synthesis, triglyceride accumulation, and lipid storage—core functions crucial for successful adipocyte differentiation [19]. By linking upstream signaling events with cellular metabolic status, SREBP1 maintains lipid homeostasis in cells during adipogenesis. Disruption of SREBP1 function leads to impaired adipocyte differentiation and lipid metabolism, emphasizing its central role in maintaining adipogenic homeostasis [20–22]. Therefore, elucidating the regulatory mechanisms upstream of SREBP1 is vital for understanding MSC adipogenesis and identifying novel targets for modulating metabolic diseases.

In this study, we aimed to investigate the role of GIPC2 in regulating adipogenic differentiation of human umbilical cord-derived MSCs (UC-MSCs) and explore the molecular mechanisms. Our findings showed that GIPC2 directly interacts with pyruvate kinase M2 (PKM2) via its PDZ domain, promoting the nuclear translocation of PKM2. This nuclear localization facilitates the activation of SREBP1, thereby enhancing the adipogenic program. Furthermore, we also showed that GIPC2 promotes adipogenesis in MSCs through the PKM2–SREBP1 signaling axis. These results provide new mechanistic insights into MSC differentiation and open promising avenues for therapeutic strategies targeting adipogenesis in the context of regenerative medicine and metabolic disorders.

## Results

### GIPC2 expression is positively correlated with adipogenic differentiation potential of MSCs

**Fig. 1:**
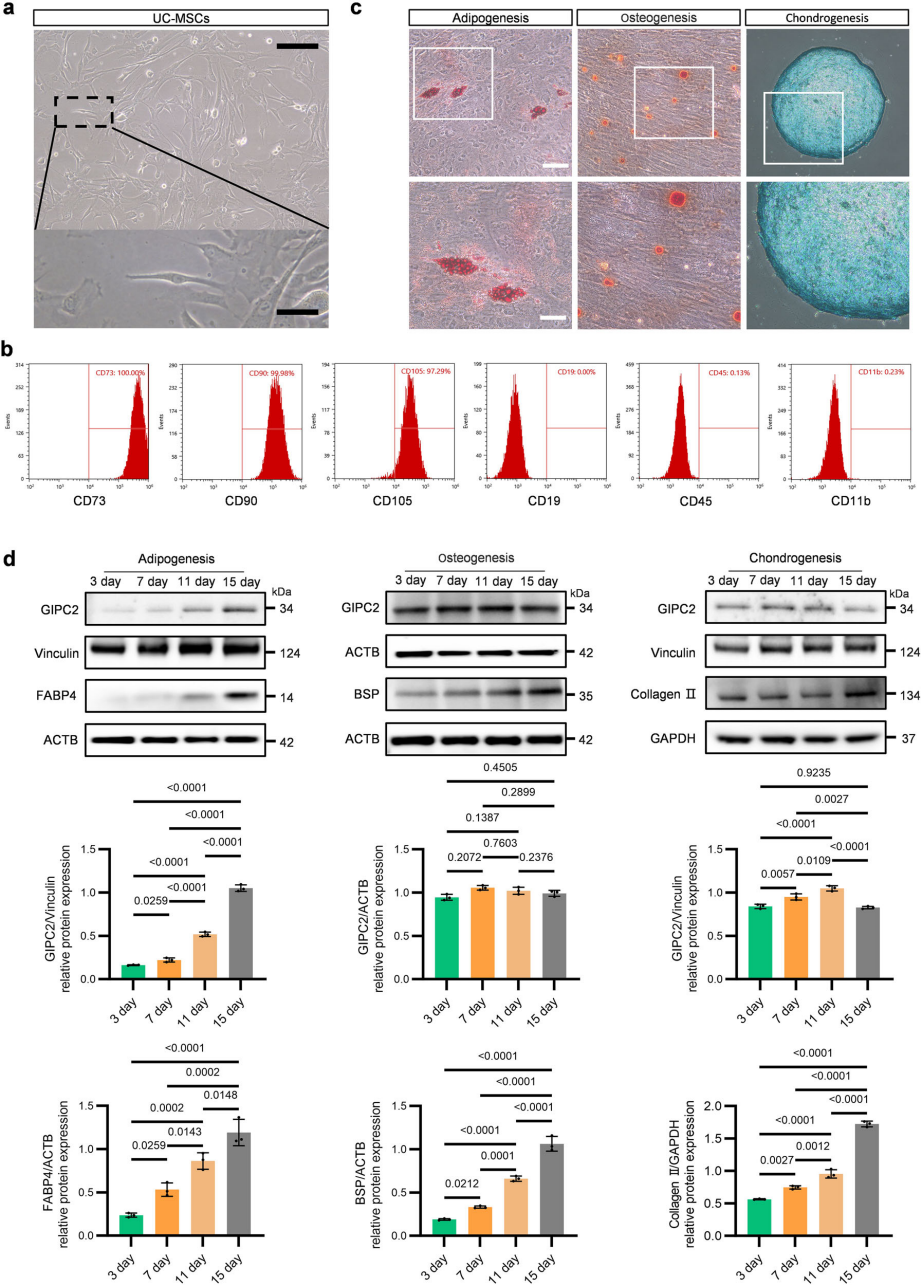
Characterization and differentiation analysis of mesenchymal stem cells (MSCs). **a** Representative images showing the morphology of MSCs. **b** Flow cytometric analysis for the identification of MSC-specific markers. **c** Staining to confirm the differentiation potential of MSCs. **d** WB analysis demonstrating the differentiation status of MSCs. All experiments were conducted in triplicate unless otherwise indicated. Statistical analysis: one-way ANOVA. Error bars: mean ± standard error of the mean (s.e.m.).

The UC-MSCs used in this study were confirmed to meet the criteria established by the International Society for Cellular Therapy (ISCT) through comprehensive characterization. Morphologically, UC-MSCs predominantly exhibited a fibroblast-like appearance under light microscopy, consistent with their characteristic growth pattern (Fig. 1a). Flow cytometry analysis further confirmed their surface antigen profile, showing ≥90% positivity for CD73, CD90, and CD105 and ≤5% positivity for CD19, CD45, and CD11b, thereby fulfilling the ISCT-defined criteria for MSC identification (Fig. 1b). To validate their multipotent differentiation capacity—a hallmark of MSCs under the ISCT guidelines—UC-MSCs were induced to differentiate into adipogenic, osteogenic, and chondrogenic lineages. Following induction, lineage-specific staining verified successful differentiation. Oil Red O (ORO) staining revealed the presence of intracellular lipid droplets, indicative of adipogenesis. Alizarin Red staining identified mineralized nodules characteristic of osteogenesis, while Alcian Blue staining showed extracellular matrix deposition, confirming chondrogenesis (Fig. 1c). These findings collectively verify that the UC-MSCs used in this study met international standards with respect to morphology, surface marker expression, and trilineage differentiation potential.

Consistent with the staining results, western blot (WB) analysis revealed the upregulation of lineage-specific markers during the differentiation process. Expression of FABP4 (adipogenic), IBSP (osteogenic), and collagen II (chondrogenic) significantly increased after 15 days of induction. Furthermore, GIPC2 expression increased progressively and consistently during adipogenic differentiation but remained unchanged during osteogenic and chondrogenic differentiation (Fig. 1d). These findings suggest that GIPC2 expression is positively associated with the adipogenic differentiation of UC-MSCs, while it appears to play a negligible role in osteogenesis or chondrogenesis. These findings highlight a potential lineage-specific function of GIPC2 in adipogenesis and underscores the need for further investigation into its underlying regulatory mechanisms.

### GIPC2 positively regulates adipogenic differentiation in MSCs

**Fig. 2:**
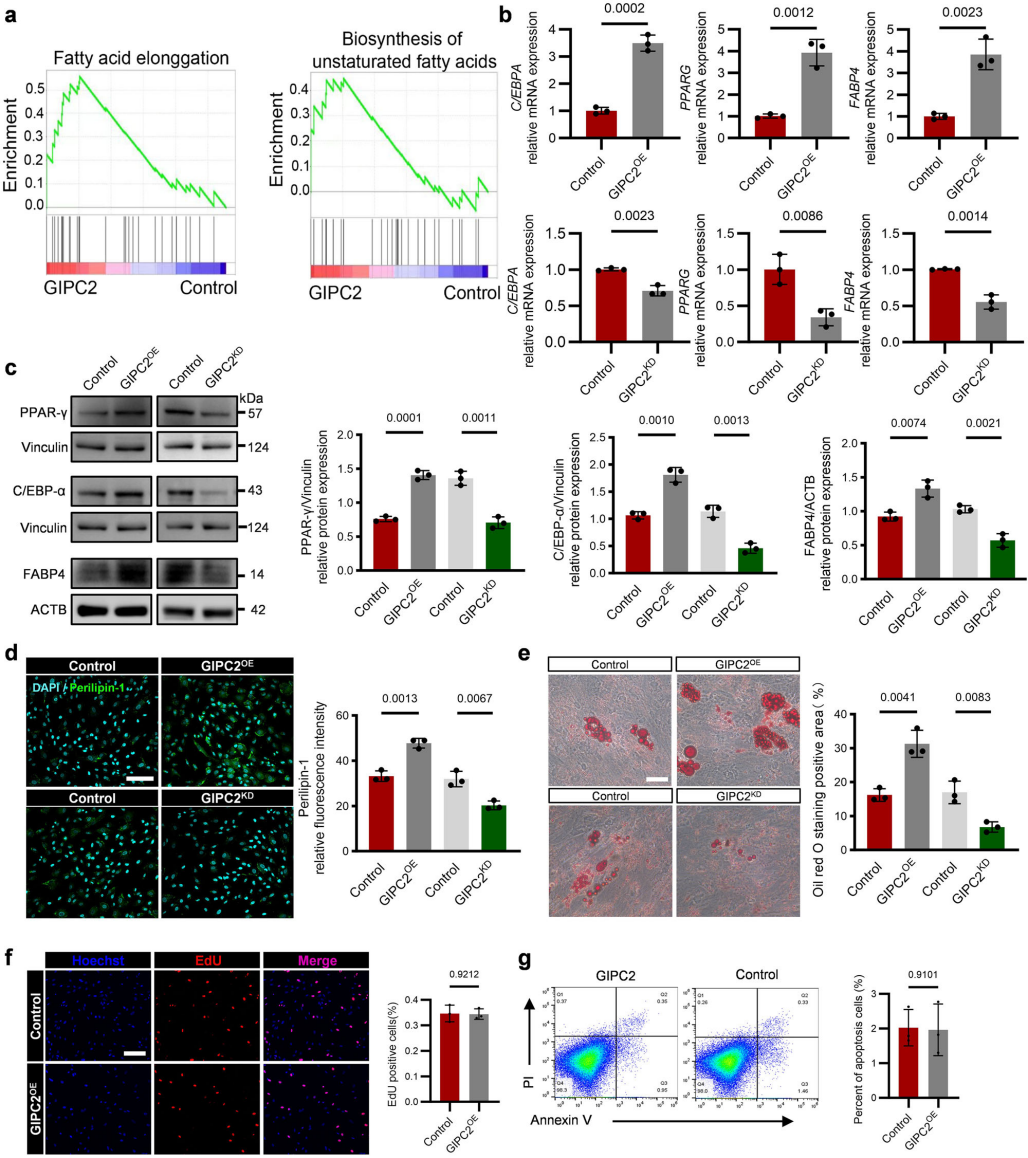
GIPC2 increases the adipogenic differentiation of MSCs without affecting their proliferation or apoptosis. **a** Pathways associated with adipogenic potential in relation to GIPC2 expression identified using Gene Set Enrichment Analysis (GSEA). **b** Transcript levels of the adipogenic markers *PPARG*, *C/EBPΑ*, and *FABP4* in UC-MSCs with GIPC2 overexpression or knockdown assessed using qRT-PCR. **c** Protein levels of the adipogenic markers PPAR-γ, C/EBP-α, and FABP4 in cells with GIPC2 overexpression or knockdown assessed using WB analysis. **d** Expression of perilipin-1 evaluated using immunofluorescence staining in UC-MSCs with GIPC2 overexpression or knockdown. **e** Visualization of lipid droplet formation during trilineage differentiation of UC-MSCs with GIPC2 overexpression or knockdown obtained using Oil Red O staining (ORO) staining. **f** Analysis of UC-MSCs proliferation in the context of GIPC2 overexpression assessed using EdU staining. **g** Flow cytometry results demonstrating UC-MSCs apoptosis under GIPC2 overexpression. Transcriptomic analysis was performed on three samples per group (*n* = 3) in (**a**). All experiments were conducted in triplicate unless otherwise indicated. Statistical analysis: For the data in (**b**–**g**), a two-sided *t*-test was used if the normality criteria were met; otherwise, the two-sided Mann‒Whitney *U* test was applied. Error bars: mean ± s.e.m.; Scale bar, 100 μm (**d**, **f**), 50 μm (**e**); **a**–**g** “GIPC2^OE/KD^” indicates UC-MSCs with GIPC2 overexpression or knockdown.

To investigate the functional role of GIPC2 in MSC adipogenesis, a lentiviral vector was constructed to modulate its expression. Transcriptomic profiling of UC-MSCs overexpressing GIPC2 revealed its active involvement in adipogenic differentiation (Fig. 2a). Quantitative real-time polymerase chain reaction (qRT-PCR) analysis showed that GIPC2 overexpression significantly upregulated key adipogenic transcription factors and markers, including *C/EBPA*, *PPARG*, and *FABP4* (Fig. 2b), whereas knockdown of GIPC2 decreased the expression of these adipogenesis-associated genes. WB further validated the corresponding trends at the protein level (Fig. 2c). Moreover, immunofluorescence staining for perilipin-1, a lipid droplet marker, confirmed these results, which aligns with the findings from qRT-PCR analyses and WB analyses (Fig. 2d). ORO staining further demonstrated a marked increase in lipid droplet accumulation in MSCs overexpressing GIPC2 during adipogenic induction. In contrast, GIPC2 knockdown led to a reduction in lipid accumulation (Fig. 2e). To assess whether this regulatory role of GIPC2 is conserved across different MSC lineages, parallel experiments were conducted in cord blood-derived (CB-MSCs), bone marrow-derived (BM-MSCs), and adipose-derived MSCs (AD-MSCs). In all tested MSC subtypes, GIPC2 overexpression promoted adipogenic differentiation, while knockdown suppressed it. This regulatory effect was not observed during osteogenic or chondrogenic differentiation (Supplementary Figs. 1–4).

To further evaluate the physiological relevance of GIPC2 in adipose tissue formation, we employed a 3D adiposphere model. Human preadipocytes embedded in Matrigel and cultured under ultra-low attachment conditions for 14 days with modulated GIPC2 expression exhibited enhanced adipose tissue morphogenesis upon GIPC2 overexpression. This effect appeared to be mediated through synergistic enhancement of both adipogenic differentiation and intercellular junction formation (Supplementary Figs. 5a–f and 6a–b). These findings strongly support the role of GIPC2 as a positive regulator of MSC adipogenic differentiation.

To determine whether the observed adipogenic effects of GIPC2 are associated with alterations in MSC proliferation or apoptosis, we performed additional functional assays. The results indicated that GIPC2 overexpression had no significant effect on either proliferation or apoptosis across experimental groups (Fig. 2f, g). These findings suggest that GIPC2 enhances adipogenic differentiation independently of its effects on cell proliferation or survival, highlighting its specific role in lineage commitment and differentiation.

### GIPC2 interacts with PKM2 via its PDZ domain

**Fig. 3:**
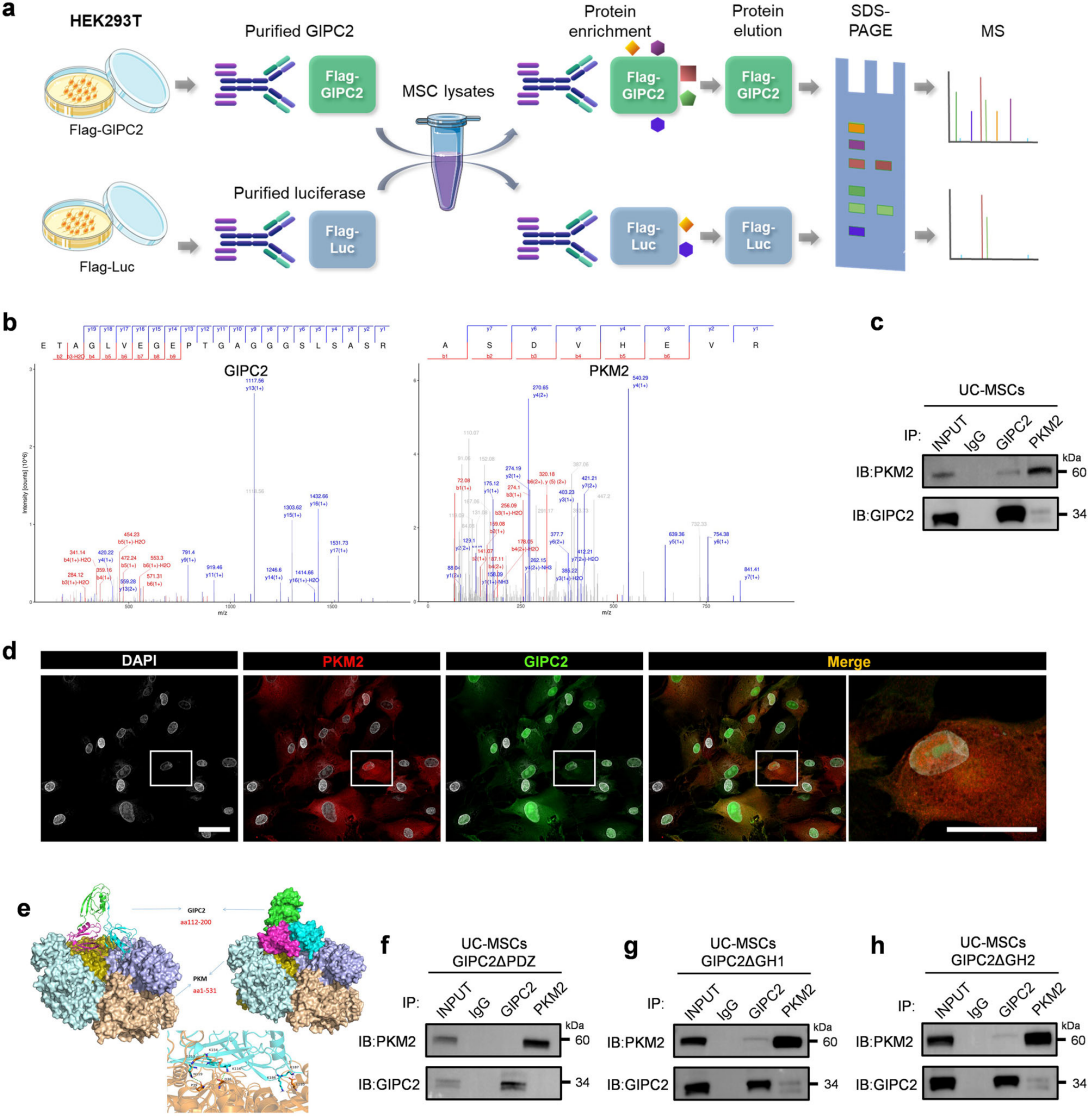
GIPC2 interacts with PKM2 via its PDZ domain. **a** Schematic diagram of Co-immunoprecipitation (Co-IP) assay followed by mass analysis. **b** Mass spectrometry (MS) analysis identified PKM2 as a potential interacting partner of GIPC2. **c** Interaction between GIPC2 and PKM2 assessed using Co-IP assays. **d** Colocalization of GIPC2 and PKM2 in MSCs revealed through immunofluorescence staining. **e** Protein structure prediction and molecular docking analysis performed to evaluate the binding affinity between PKM2 and GIPC2. **f**–**h** Interaction between **f** PKM2 and GIPC2 (deletion mutant of GIPC2△PDZ), **g** PKM2 and GIPC2 (deletion mutant of GIPC2△GH1), and **h** PKM2 and GIPC2 (deletion mutant of GIPC2△GH2) assessed using Co-IP assays. Error bars: mean ± s.e.m.; Scale bar, 25 μm (**d**).

To elucidate the mechanism by which GIPC2 regulates adipogenesis, we performed co-immunoprecipitation (Co-IP) and mass spectrometry (MS) analyses to identify GIPC2-interacting proteins (Fig. 3a). Several candidate proteins were identified, among which PKM2 was found within the Co-IP complex, suggesting a direct interaction between GIPC2 and PKM2 (Fig. 3b). To validate this interaction, we co-expressed HA-tagged PKM2 and FLAG-tagged GIPC2 in UC-MSCs. Co-IP assays subsequently confirmed that PKM2 co-immunoprecipitated with GIPC2 (Fig. 3c). Additionally, immunofluorescence staining revealed colocalization of GIPC2 and PKM2 in UC-MSCs (Fig. 3d), further supporting a physical and functional association between these two proteins.

To investigate the structural basis of the GIPC2–PKM2 interaction, molecular docking simulations were conducted. These analyses predicted that the PDZ domain of GIPC2—one of three domains including GH1, GH2, and PDZ—serves as the primary interface for PKM2 binding based on optimal structural complementarity and binding energetics (Fig. 3e). To validate this prediction experimentally, we generated FLAG-tagged GIPC2 deletion constructs (ΔPDZ, ΔGH1, and ΔGH2) and HA-tagged PKM2 deletion mutants (ΔN + A1, ΔB, ΔA2, and ΔC). Co-IP assays in UC-MSCs revealed that deletion of the PDZ domain (GIPC2ΔPDZ) completely abolished the interaction with PKM2, whereas deletion of either the GH1 or GH2 domains did not impair binding (Fig. 3f–h). Complementary analyses using PKM2 deletion constructs showed that removal of either the N-terminal domain (PKM2ΔN + A1) or the C-terminal domain (PKM2ΔC) disrupted binding to GIPC2. In contrast, deletion of the B/A2 domains (PKM2ΔB/A2) did not affect the interaction (Supplementary Fig. 7a). These findings collectively indicate that the interaction between GIPC2 and PKM2 is mediated through the PDZ domain of GIPC2 and requires intact N- and C-terminal domains of PKM2.

### GIPC2 promotes adipogenic differentiation of MSCs by facilitating nuclear translocation of PKM2

**Fig. 4:**
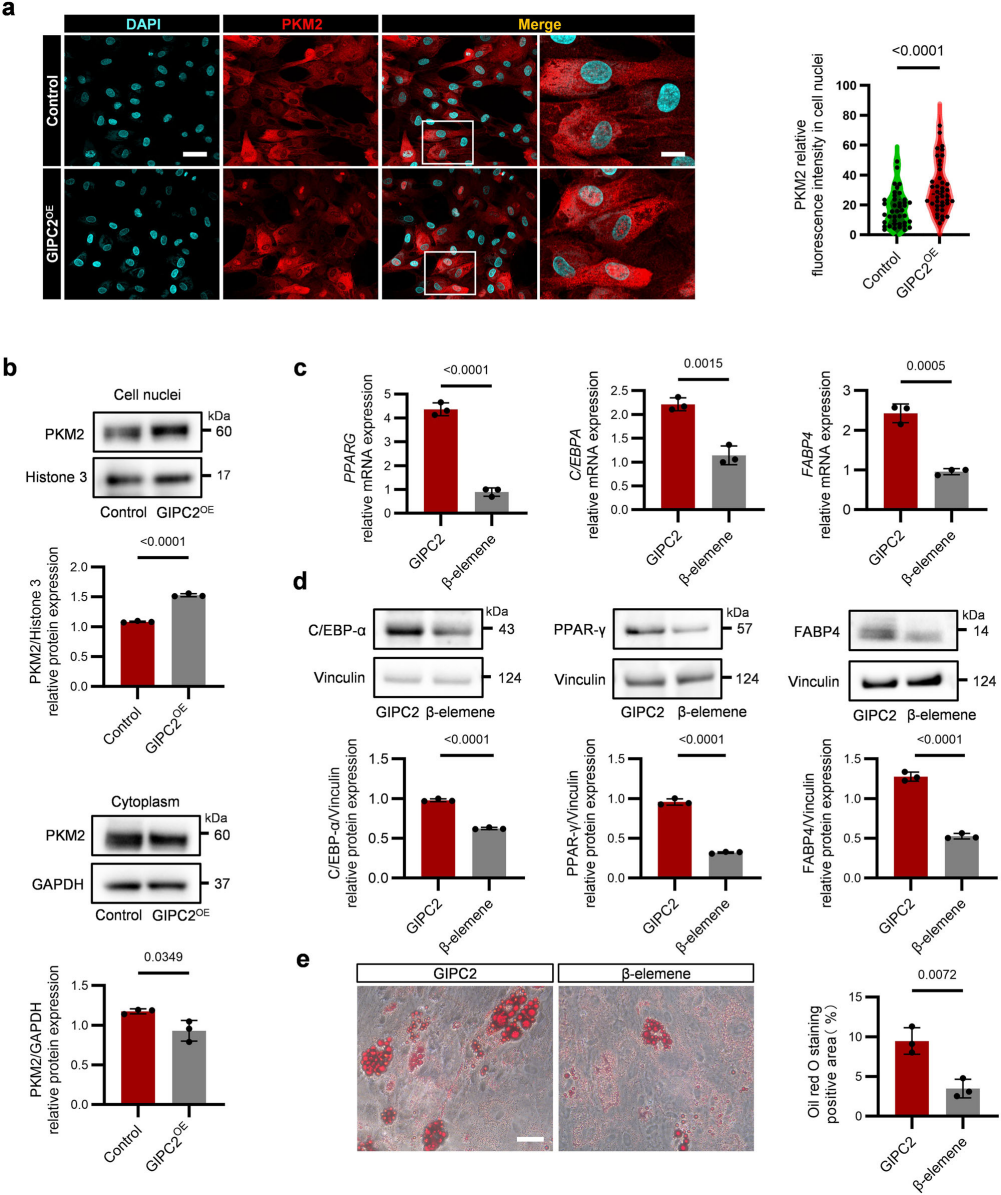
GIPC2 promotes the nuclear translocation of PKM2 to increase the adipogenic differentiation of UC-MSCs. **a** Nuclear localization of PKM2 in GIPC2-overexpressing MSCs assessed using immunofluorescence analysis. **b** Evaluation of PKM2 levels in the nuclear and cytoplasmic fractions of GIPC2-overexpressing MSCs using WB analysis. **c** Transcript levels of the adipogenic markers *PPARG*, *C/EBPΑ*, and *FABP4* in GIPC2-overexpressing UC-MSCs with or without β-elemene treatment assessed using qRT-PCR. **d** Protein levels of the adipogenic markers PPAR-γ, C/EBP-α, and FABP4 in GIPC2-overexpressing UC-MSCs with or without β-elemene treatment assessed using WB analysis. **e** Visualization of lipid droplet formation in GIPC2-overexpressing UC-MSCs treated with β-elemene following 15 days of adipogenic induction obtained using ORO staining. All experiments were conducted in triplicate unless otherwise indicated. Statistical analysis: For the data in (**a**–**e**), a two-sided *t*-test was used if the normality criteria were met; otherwise, the two-sided Mann‒Whitney *U* test was applied. Within each replicate, we analyzed ≥ 50 randomly selected cells across multiple imaging fields (**b**). Error bars: mean ± s.e.m.; Scale bar, 25 μm (**a**), 10 μm (**a**, enlarged image), 50 μm (**e**). **a**, **b** “GIPC2” indicates MSCs overexpressing GIPC2, and “Control” indicates MSCs not overexpressing GIPC2. **c**–**e** “GIPC2” indicates MSCs overexpressing GIPC2, and “β-elemene” indicates GIPC2-overexpressing MSCs with β-elemene.

To investigate the mechanism by which GIPC2 promotes adipogenic differentiation in MSCs, we first examined the subcellular localization of PKM2 in GIPC2-overexpressing cells using immunofluorescence staining. The results revealed a marked enrichment of PKM2 in the nuclei of MSCs overexpressing GIPC2 (Fig. 4a and Supplementary Fig. 8a–c). In contrast, the nuclear-to-cytoplasmic ratio of GIPC2 itself remained unchanged following overexpression and (Supplementary Fig. 8d), suggesting that GIPC2 does not translocate but rather facilitates the nuclear import of PKM2 during adipogenic differentiation. To validate these findings, we performed nuclear and cytoplasmic fractionation of GIPC2-overexpressing and control UC-MSCs. WB analysis revealed reduced cytoplasmic and increased nuclear levels of PKM2 in GIPC2-overexpressing cells compared to that in control cells (Fig. 4b and Supplementary Fig. 8e–g). Critically, GIPC2 perturbation did not alter endogenous PKM2 expression at either transcriptional or protein levels. Conversely, neither PKM2 overexpression nor knockdown measurably affected GIPC2 expression (Supplementary Fig. 9a–d). These findings support the hypothesis that GIPC2 enhances adipogenesis by promoting the nuclear translocation of PKM2.

To further confirm the functional relevance of this mechanism, we treated GIPC2-overexpressing UC-MSCs with β-elemene, a known inhibitor of PKM2 nuclear import [23], and assessed PKM2 localization via immunofluorescence. The results revealed that β-elemene treatment effectively blocked the nuclear accumulation of PKM2 in GIPC2-overexpressing cells (Supplementary Fig. 10a). The effect of this inhibition on adipogenic differentiation was then assessed through qRT-PCR and WB analysis of adipogenic markers (Fig. 4c, d). The results revealed significantly reduced expression of key adipogenic markers in β-elemene-treated cells, indicating impaired adipogenic differentiation capacity of GIPC2-overexpressing cells.

To evaluate the effect of β-elemene on lipid droplet formation during adipogenesis, we conducted ORO staining after 15 days of adipogenic induction. GIPC2-overexpressing cells treated with β-elemene exhibited a significantly lower number of lipid droplets than the untreated GIPC2-overexpressing UC-MSCs (Fig. 4e). This reduction in lipid accumulation underscores the critical function of PKM2 nuclear translocation in GIPC2-mediated adipogenic differentiation.

To elucidate the molecular mechanism underlying GIPC2-mediated PKM2 nuclear import, we examined phosphorylation of PKM2 at Ser37, a known prerequisite for its nuclear translocation. Our data demonstrated that the PDZ domain of GIPC2, which mediates its interaction with PKM2, facilitates Ser37 phosphorylation. In GIPC2-overexpressing UC-MSCs, deletion of the PDZ domain in GIPC2 significantly reduced Ser37 phosphorylation and attenuated nuclear accumulation of phosphorylated PKM2 (Supplementary Fig. 11a, b). In 3D adipospheres, deletion of the PDZ domain in GIPC2 also consequently restored the metabolic enzyme activity and inhibited lipogenesis (Supplementary Fig. 11c–g). These results indicate that GIPC2 promotes the nuclear import of PKM2 through a phosphorylation-dependent mechanism involving its PDZ domain.

Collectively, these findings demonstrate that GIPC2 enhances the adipogenic differentiation capacity of UC-MSCs by facilitating PKM2 nuclear translocation. This nuclear localization of PKM2, mediated by PDZ-dependent phosphorylation at Ser37, is essential for the pro-adipogenic function of GIPC2 and highlights its critical role in MSC lineage commitment.

### GIPC2 promotes adipogenic differentiation of MSCs via the PKM2-SREBP1 axis

**Fig. 5:**
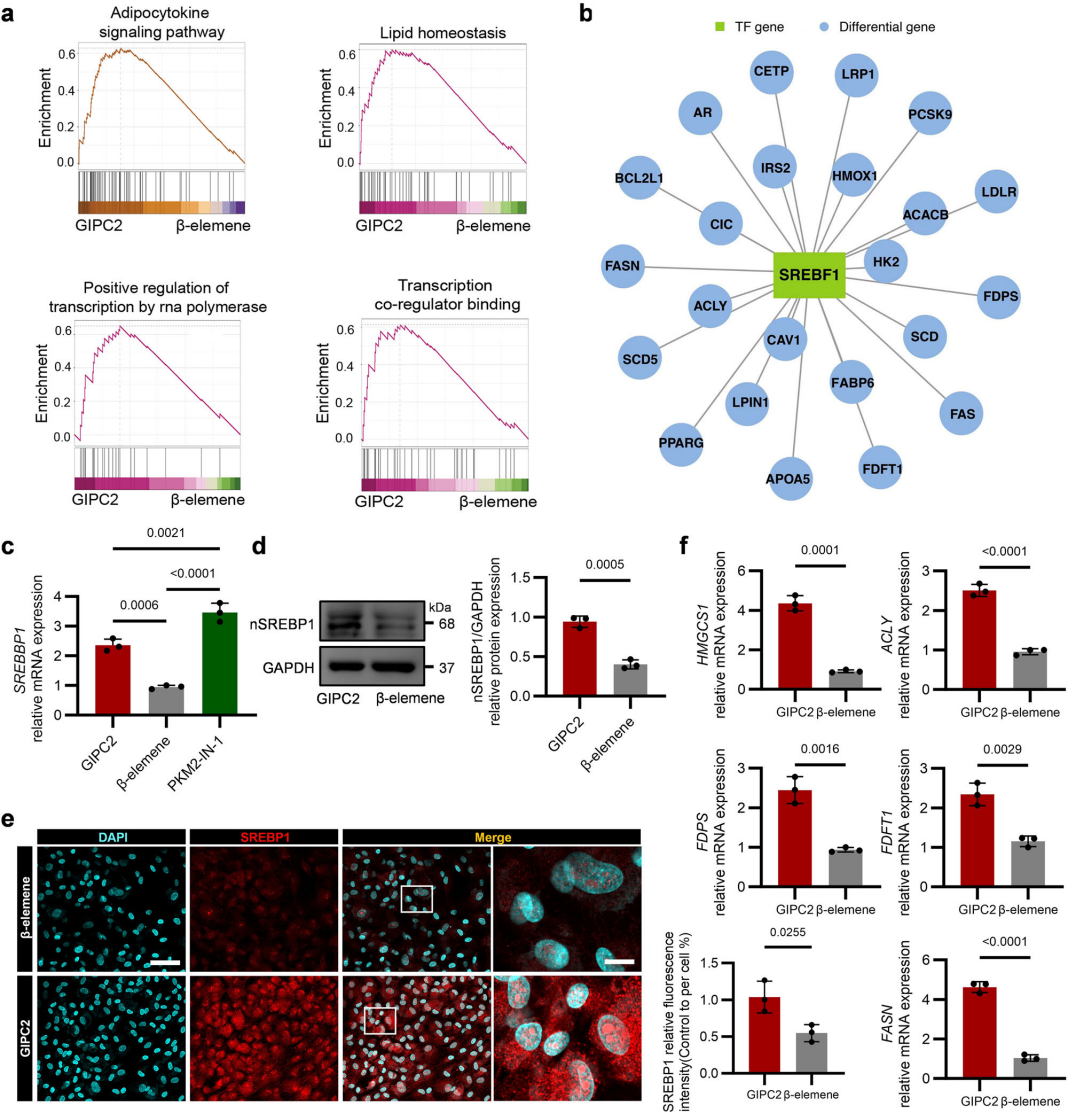
GIPC2 regulates SREBP1 activity. **a** GSEA comparing gene sets involved in the adipocytokine signaling pathway, lipid homeostasis, positive regulation of transcription by RNA polymerase and transcription coregulator binding between GIPC2-overexpressing UC-MSCs treated with or without β-elemene after 7 days of adipogenic induction. **b** Network plot illustrating transcription factors (TFs) enriched with differentially expressed genes (DEGs) in GIPC2-overexpressing UC-MSCs treated with or without β-elemene, with green nodes representing TFs and blue nodes representing target DEGs. **c** qRT-PCR analysis of *SREBP1* in GIPC2-overexpressing UC-MSCs following β-elemene or PKM2-IN-1 treatment. **d**, **e** WB and immunofluorescence analyses evaluating SREBP1 expression in GIPC2-overexpressing UC-MSCs following β-elemene treatment. **f** qRT-PCR analysis of *ACLY, FASN, FDPS, FDFT1*, and *HMGCS1* in GIPC2-overexpressing UC-MSCs following β-elemene treatment. Transcriptomic analysis was performed on three samples per group (*n* = 3) in (**a**). All experiments were conducted in triplicate unless otherwise indicated. Statistical analysis: one-way ANOVA (**c**), two-sided *t*-test (**d**–**f**). Error bars: mean ± s.e.m.; Scale bar, 100 μm (**e**), 10 μm (**e**, enlarged image). **a**, **d**–**f** “GIPC2” indicates UC-MSCs overexpressing GIPC2, and “β-elemene” indicates GIPC2-overexpressing UC-MSCs with β-elemene. **c** “GIPC2” indicates UC-MSCs overexpressing GIPC2, “β-elemene” indicates GIPC2-overexpressing UC-MSCs with β-elemene, and “PKM2-IN-1” indicates GIPC2-overexpressing MSCs with PKM2-IN-1.

To investigate the mechanism by which PKM2 nuclear translocation contributes to adipogenic differentiation, we performed transcriptomic sequencing of GIPC2-overexpressing UC-MSCs treated with or without the PKM2 nuclear import inhibitor β-elemene. The analysis revealed that inhibiting PKM2 nuclear translocation significantly disrupted several key biological processes and pathways critical for adipogenesis, including the adipocytokine signaling pathway, lipid homeostasis, and transcriptional regulation (Fig. 5a). These findings suggest that PKM2 nuclear translocation actively regulates the transcriptional and metabolic pathways essential for MSC adipogenic differentiation. Once inside the nucleus, PKM2 likely acts beyond its metabolic function, modulating transcriptional networks and activating key signaling cascades, underscoring its dual role as both a metabolic enzyme and a nuclear regulator under the regulatory influence of GIPC2.

To identify transcription factors affected by PKM2 nuclear translocation, we predicted transcriptional activity based on differentially expressed genes (DEGs) from the transcriptomic data. Among the predicted transcription factors, *SREBP1* emerged as a key transcriptional regulator specifically associated with adipogenesis (Fig. 5b). The activation of SREBP1 implies that nuclear PKM2 may modulate transcription factor activity, thereby amplifying downstream regulatory networks critical for adipogenic differentiation. This is consistent with the observed enrichment of transcription-related pathways and positions SREBP1 as a likely mediator of PKM2-dependent transcriptional regulation.

Subsequent validation confirmed the role of SREBP1 in GIPC2-mediated adipogenesis via PKM2 nuclear translocation. qRT-PCR analysis revealed that *SREBP1* transcription was significantly decreased in GIPC2-overexpressing cells treated with β-elemene. In contrast, treatment with the PKM2 nuclear translocation activator PKM2-IN-1 (compound 3k) markedly increased *SREBP1* transcription relative to untreated GIPC2-overexpressing cells (Fig. 5c and Supplementary Fig. 12a).

WB and immunofluorescence staining further confirmed increased nuclear accumulation of SREBP1 in GIPC2-overexpressing cells compared with β-elemene-treated cells (Fig. 5d, e). Moreover, transcriptional analysis of *SREBP1* target genes demonstrated that SREBP1 activity was significantly enhanced in the absence of β-elemene (Fig. 5f), highlighting its key regulatory role during adipogenic differentiation. Additionally, pharmacological inhibition of PKM2 enzymatic activity using either Shikonin (which disrupts PKM2 oligomerization non-selectively) or PKM-IN-1 (which stabilizes the dimeric form known to translocate to the nucleus and regulate transcription) produced contrasting effects on adipogenesis [24–26]. In contrast, PKM2 knockdown reduced lipid accumulation and impaired adipogenic differentiation in UC-MSCs. These findings underscore the critical importance of PKM2’s transcriptional regulatory role, rather than its metabolic enzyme activity, within this pathway (Supplementary Figs. 12c, d and 13a, c, e). Collectively, these findings indicate that GIPC2 promotes adipogenesis in MSCs by enhancing SREBP1 activity through PKM2 nuclear translocation. This establishes the PKM2–SREBP1 axis as a critical transcriptional and metabolic regulatory mechanism in MSC adipogenic commitment.

**Fig. 6:**
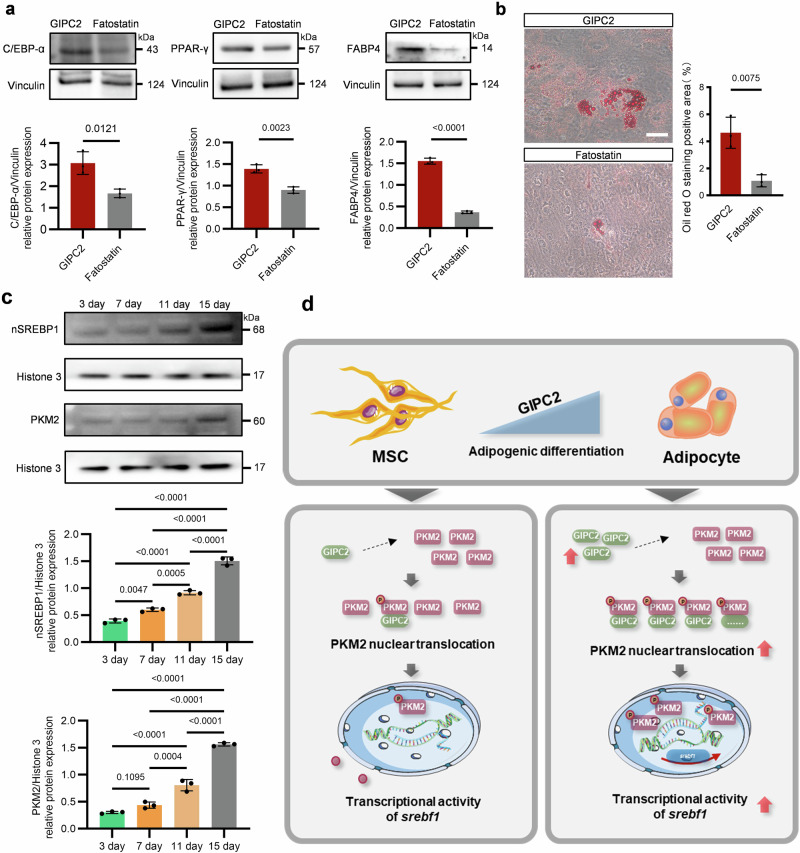
GIPC2 promotes adipogenic differentiation in MSCs via the PKM2-SREBP1 axis. **a** WB analysis of the adipogenic markers PPAR-γ, C/EBP-α, and FABP4 in GIPC2-overexpressing MSCs treated with Fatostatin. **b** ORO staining of Fatostatin-treated GIPC2-overexpressing UC-MSCs to assess lipid droplet formation after 15 days of adipogenic induction. **c** WB analysis of nuclear proteins isolated from UC-MSCs undergoing adipogenic differentiation for 15 days was performed to assess the nuclear expression of SREBP1 and PKM2 during the differentiation process. **d** Mechanism schematic diagram of GIPC2 promoting adipogenic differentiation in MSCs via the PKM2-SREBP1 axis. Statistical analysis: one-way ANOVA (**c**), two-sided *t*-test (**a**, **b**). Error bars: mean ± s.e.m.; Scale bar, 50 μm (**b**). **a**, **b** “GIPC2” indicates UC-MSCs overexpressing GIPC2, and “Fatostatin” indicates GIPC2-overexpressing MSCs with Fatostatin.

To further validate SREBP1 as a downstream effector of GIPC2, we treated GIPC2-overexpressing MSCs with Fatostatin, a specific inhibitor of SREBP1. WB analysis showed a marked reduction in the expression of key adipogenic markers—FABP4, C/EBP-α, and PPAR-γ—in Fatostatin-treated GIPC2-overexpressing cells (Fig. 6a). Consistent with this, ORO staining confirmed that Fatostatin treatment significantly impaired lipid droplet formation (Fig. 6b). Similarly, SREBP1 knockdown in UC-MSCs attenuated adipogenic differentiation (Supplementary Fig. 13b, d, f), reinforcing the central role of SREBP1 as a downstream mediator of GIPC2 activity.

To investigate the temporal dynamics of PKM2 and SREBP1 during differentiation, we quantified their nuclear expression at various stages of adipogenesis (Fig. 6c). Both PKM2 and SREBP1 exhibited progressively increased nuclear localization over time, which closely paralleled the advancement of adipogenic differentiation. This dynamic expression pattern aligns with prior findings that GIPC2 expression also increases during differentiation, suggesting that GIPC2 upregulation may drive PKM2 nuclear accumulation and subsequently activate SREBP1 (Fig. 6d and Supplementary Fig. 14a). These results provide further evidence for the cooperative function of PKM2 and SREBP1 in mediating MSC adipogenesis.

Taken together, our findings establish the GIPC2–PKM2–SREBP1 axis as a central regulatory pathway in MSC adipogenic differentiation (Supplementary Fig. 13g–i). Together with above-mentioned results demonstrating that GIPC2 promotes–PKM2 nuclear translocation and increases SREBP1 activation (Supplementary Figs. 8h, i, 12b, and 14b, c). This mechanism highlights the potential of targeting GIPC2 and PKM2 for therapeutic modulation of adipogenesis and related metabolic processes.

## Discussion

The GIPC family comprises highly conserved scaffolding proteins initially identified through their interaction with the G protein-coupled receptor regulator GAIP, highlighting their role as mediators of intracellular signaling pathways. This family includes three isoforms—GIPC1, GIPC2, and GIPC3—each characterized by a conserved PDZ domain that facilitates interactions with a wide array of signaling molecules. Through these interactions, GIPCs participate in diverse cellular processes, including membrane trafficking, cytoskeletal organization, and signal transduction [27]. Among these isoforms, GIPC2 has been most extensively studied in the context of tumor biology, where it has been implicated in regulating key processes such as EMT, metabolic reprogramming, and cell survival [1, 28–30]. These pathways, which are fundamental to oncogenesis, also overlap mechanistically with those involved in stem cell plasticity and lineage commitment, suggesting a broader physiological role for GIPC2 in modulating cell fate decisions. Despite its well-characterized functions in cancer, the role of GIPC2 in stem cell biology remains largely unexplored [31]. Previous studies have reported a decline in GIPC2 expression during the in vitro expansion of dental pulp stem cells; however, GIPC2 silencing did not affect their osteogenic differentiation potential [32]. In contrast to these findings, the present study is the first to define a direct and functional role for GIPC2 in MSC differentiation, specifically in adipogenesis. By elucidating the role of GIPC2 in modulating the PKM2–SREBP1 signaling axis, we demonstrate that GIPC2 serves as a critical regulator of adipogenic differentiation. These findings not only broaden the biological importance of GIPC2 beyond oncology but also highlight its versatility as a key regulator of stem cell differentiation and lineage commitment.

PKM2 exhibits dual functionality: in the cytoplasm, its tetrameric form catalyzes the final step of glycolysis, whereas in the nucleus, its dimeric form functions as a transcriptional co-regulator that promotes lipogenesis [33, 34]. Recent studies have demonstrated that dimerization of PKM2 leads to the activation of SREBP1, thereby enhancing adipogenesis [35]. In stem cells, PKM2 conformational states also regulate adipogenic differentiation; for instance, Cen et al. (2020) reported that enforced high-activity PKM2 (tetrameric state) inhibits adipogenesis in MSCs [36]. Considering these, our study newly identifies GIPC2 as a critical upstream regulator of PKM2 conformation and function during MSC adipogenesis. Specifically, we show that GIPC2 promotes PKM2 phosphorylation at Ser37 via its PDZ domain, thereby facilitating PKM2 dimerization and nuclear translocation. This mechanism reveals a previously uncharacterized pathway by which GIPC2 orchestrates the metabolic–transcriptional coupling mediated by PKM2. To elucidate the structural basis of this regulation, we demonstrated that GIPC2 directly binds PKM2 through its PDZ domain. This interaction is essential for facilitating the nuclear translocation of PKM2 and is a prerequisite for its non-glycolytic role as a transcriptional coactivator [37–39]. Once localized to the nucleus, PKM2 activates SREBP1, a master transcription factor governing lipid biosynthesis and adipocyte maturation [40]. While the metabolic and transcriptional functions of PKM2 have been previously characterized in various contexts, our findings uniquely position GIPC2 as a crucial upstream modulator of the nonenzymatic activity of PKM2 in MSCs [36, 41]. Unlike prior studies focusing solely on the intrinsic regulatory properties of PKM2, we demonstrate that its nuclear localization—and thus its transcriptional activity—is contingent upon GIPC2-mediated signaling. Accordingly, we establish a direct GIPC2–PKM2 regulatory axis [42]. These findings highlight a novel role for GIPC2 in linking metabolic enzyme regulation to transcriptional programming during stem cell differentiation. By acting as a facilitator of PKM2-SREBP1 signaling, GIPC2 may serve as a potential therapeutic target for modulating adipogenic differentiation and treating metabolic disorders. Collectively, this work not only expands the functional repertoire of GIPC2 but also provides a new framework for understanding its broader involvement in regulating cell fate decisions.

MSCs are central to regenerative medicine due to their multipotent capacity to differentiate into various lineages, including adipocytes. Adipogenic differentiation is particularly critical for applications such as soft tissue regeneration, autologous fat grafting, and the treatment of metabolic disorders, including lipodystrophy [43, 44]. In this study, we identified the GIPC2–PKM2–SREBP1 signaling axis as a novel regulatory pathway governing the adipogenic differentiation of MSCs, specifically in UC-MSCs in vitro. This discovery significantly advances our understanding of the molecular mechanisms underlying UC-MSC adipogenesis and provides the first mechanistic framework for optimizing MSC-based therapies. Although our investigation focused on UC-MSCs under in vitro conditions, the elucidated signaling axis offers valuable insights into the regulation of adipogenic differentiation in other MSC subtypes. These findings may inform strategies to modulate endogenous MSCs within their native microenvironments to enhance tissue repair and regeneration [45]. Furthermore, defining the molecular basis of this regulatory pathway opens new avenues for therapeutic innovation—such as the development of small molecules or biologics designed to promote GIPC2–PKM2 interaction, thereby enhancing adipogenic potential. Overall, our findings address a critical gap in the understanding of MSC lineage commitment and differentiation. By establishing a functional and mechanistic role for the GIPC2–PKM2–SREBP1 axis, this work lays the groundwork for expanding the translational and clinical applications of MSCs in regenerative medicine and related fields [46].

While our findings reveal a novel regulatory mechanism, further studies are required to comprehensively elucidate the broader implications of the GIPC2–PKM2–SREBP1 axis in MSC adipogenesis. A primary limitation of this study is its exclusive focus on UC-MSCs. Although the adipogenic potential of UC-MSCs is well established, it remains unclear whether this regulatory axis is conserved in MSCs derived from other tissue sources, such as bone marrow or adipose tissue [47]. Future research should investigate whether similar molecular mechanisms operate across different MSC populations and assess how variations in GIPC2 or PKM2 expression levels influence lineage commitment in these cells. Such studies could significantly expand the translational relevance of our findings and inform the development of MSC-based therapies tailored to specific tissue sources. Moreover, although we identified the GIPC2–PKM2–SREBP1 axis as a crucial pathway in adipogenic differentiation, its interactions with other key regulatory and metabolic pathways have not been fully delineated. Notably, although our preliminary findings indicate that GIPC2 regulates adipogenesis independently of the Wnt/β-catenin, TGF-β, and PI3K/AKT pathways, potential crosstalk between this axis and these signaling cascades—known to critically regulate MSC fate decisions—warrants detailed investigation [48–50]. Elucidating these interactions will contribute to a more comprehensive understanding of the complex signaling networks that govern stem cell differentiation and may reveal additional therapeutic targets for modulating MSC lineage commitment in regenerative medicine.

In summary, our study establishes GIPC2 as a novel and critical regulator of MSC adipogenic differentiation through its modulation of the PKM2–SREBP1 signaling axis. By demonstrating that GIPC2 facilitates the nuclear translocation of PKM2 and the subsequent activation of SREBP1, we uncover a previously unrecognized regulatory mechanism that links metabolic enzyme function with transcriptional control during stem cell differentiation [51]. These findings offer significant new insights into the molecular underpinnings of adipogenesis and position GIPC2 as a potential therapeutic target for modulating MSC differentiation and maintaining lipid metabolic homeostasis. This work lays the foundation for the development of targeted strategies to enhance adipogenic differentiation and provides a valuable framework for optimizing MSC-based regenerative therapies.

## Materials and Methods

### Isolation and culture of MSCs

This study has been approved by the Ethics Committee of the First Affiliated Hospital of Dalian Medical University (approval number: YJ-GXB-2022-01). The umbilical cord samples were obtained from healthy newborns delivered via cesarean section, with written informed consent provided by their parents. All procedures were conducted in accordance with the relevant guidelines and regulations. Umbilical cords were carefully dissociated into single MSCs, which were then cultured and expanded in Mesenchymal stem cell medium according to the protocols. The isolation, extraction, and expansion procedures adhered strictly to established international standards to ensure reproducibility and compliance with guidelines. The obtained MSCs were subjected to routine morphological observation and functional characterization to confirm their identity and viability, providing a reliable cellular basis for subsequent experimental analyses. CB-MSCs (OriCell®, HUXUB-01001), BM-MSCs (OriCell®, HUXMA-01001), and AD-MSCs (OriCell®, HUXMD-01001) were purchased from Cyagen Biosciences. All MSC types were expanded using serum-free MSC Expansion Kit (seafrom, AS-33) for subsequent differentiation studies.

### Human preadipocytes and 3D adiposphere culture

Primary human preadipocytes were purchased from Zhong Qiao Xin Zhou Biotechnology. Cells maintained as 2D monolayers were digested with 0.05% trypsin at 37 °C. Following centrifugation, the cell pellet was collected. Matrigel (Corning, 354277) was thawed on ice. The cell pellet was then thoroughly mixed with Matrigel to form a cell-gel suspension. This suspension was transferred into ultra-low attachment plates (NEST Biotechnology, 70201B01) using pre-chilled ultra-low attachment pipette tips. Plates were incubated at 37 °C to allow gel solidification. After solidification, complete medium for human preadipocytes (ZQXZBIO, PCM-H-123) was added and cultures were maintained for 3 days, with gentle agitation on day 2 to prevent adhesion. Upon adiposphere formation, the medium was replaced with adipogenic differentiation induction medium to initiate differentiation.

### Adipogenic, osteogenic, and chondrogenic differentiation induction

MSCs were seeded into 6-well plates (BaiDi Biotechnology Co., Ltd.(BDBIO), H803500) at a density of 1 × 10⁴ cells/cm² and allowed to adhere overnight. For adipogenic differentiation, the cells were cultured in adipose differentiation medium (Stem Cell Technologies, 05412), while osteogenic differentiation was induced via an osteogenic differentiation kit (Stem Cell Technologies, 05465). As for chondrogenic differentiation, 2 × 10⁶ MSCs were resuspended in 2 mL of chondrocyte differentiation medium (Stem Cell Technologies, 05455), and 0.5 mL of the cell suspension was transferred into each 15 mL polypropylene tube. The tubes were tightly capped and centrifuged at 1000 rpm for 10 min at 15–25 °C to allow the cells to form a pellet. After centrifugation, the lids of the tubes were slightly loosened (half-screwed) to allow gas exchange, and the tubes were placed upright in a humidified incubator for differentiation. The differentiation medium was changed every 3 days.

### Oil Red O staining

After specific induction for adipogenic differentiation, the MSCs were fixed with 4% paraformaldehyde at room temperature for 15 min to preserve their cellular morphology. The fixed cells were rinsed three times with phosphate-buffered saline (PBS, pH 7.4) to remove residual fixative. The cells were then stained with (Oil Red O) ORO solution (Sigma, O1391) for 30 min, followed by additional washes with PBS (SEVEN, SC106-01) to remove excess stain. The stained cells were observed and recorded immediately under a microscope.

### Alizarin red staining

At alizarin red staining experiments, the cells were fixed with 4% paraformaldehyde at room temperature for 15 min and then rinsed three times with PBS (pH 7.4), with each wash lasting 5 min. The fixed cells were stained with 10% Alizarin Red S solution (Sigma, A5533) for 10 min to detect calcium deposits, a hallmark of osteogenesis. Excess dye was removed by washing the cells three times with PBS. The stained mineralized nodules were observed and recorded via an optical microscope.

### Alcian blue staining

In the experiment stained with alcian blue, cartilage spheres were processed into thin sections via a cryostat microtome (Leica CM1950). The dried sections were stained with an appropriate volume of Alcian blue staining solution (Beyotime, C0155M) for 1 h at room temperature to identify glycosaminoglycans, which are key components of cartilage. After staining, the sections were rinsed under running tap water for 5 min to remove excess dye, followed by air drying. The staining results were examined under a microscope to evaluate the effectiveness of chondrogenic differentiation.

### Identification of UC-MSC surface markers via flow cytometry

To identify the surface characteristic markers of UC-MSCs, the cells were digested with pancreatic enzymes and divided into two tubes, with 4 × 10⁵ cells per tube. The cells were subsequently centrifuged at 1000 rpm for 5 min at room temperature, after which the supernatant was carefully discarded. The cell pellets were washed three times with PBS (pH 7.4) to remove residual enzymes and debris. The following antibodies, diluted according to the manufacturer’s instructions, were added to the tubes: CD73 (CST, 13160S, 1:1000), CD90 (CST, 13801S, 1:1000), CD105 (CST, 14606S, 1:1000), CD19 (CST, 3574S, 1:1000), CD11b (CST, 3621S, 1:1000), and CD45 (CST, 70257S, 1:1000). The cells were incubated with the specified antibodies at 4 °C for 30 min to ensure binding. After incubation, the cells were washed twice with PBS containing 0.5% fetal bovine serum (FBS) to remove excess unbound antibodies. The cell suspensions were then transferred to specialized detection tubes for flow cytometry analysis. The expression of MSC surface markers was assessed via flow cytometry, with a focus on the positive markers CD73, CD90, and CD105 and the negative markers CD19, CD11b, and CD45. The results were applied to proof the surface characteristic markers profile of the MSCs, ensuring that the cells met the criteria defined by the ISCT.

### Western blot

After different inductions or treatments, the MSCs then were lysed via RIPA buffer (Abcam, ab288006) at the designated time points, and the protein content of the lysates was quantified via BCA protein assay kit (Thermo Fisher Scientific, A55864). Equal amounts of protein from each sample were calculated and prepared for SDS‒PAGE. Proteins were separated electrophoretically, transferred onto PVDF membranes, and blocked with Rapid Blocking Solution at room temperature for 30 min to prevent nonspecific binding. The membranes were then washed with TBST and incubated overnight at 4 °C with the following primary antibodies, which were diluted as specified by the manufacturers: GIPC2 (Abcam, ab175272, 1:1000), PKM2 (CST, 46687, 1:1000), SREBP1 (CST, 95879, 1:1000), C/EBP-α (Abcam, ab40764, 1:1000), FABP4 (Abcam, ab92501, 1:2500), PPAR-γ (CST, 2443, 1:1000), SOX-9 (Abcam, ab185966, 1:5000), Collagen II (Proteintech, 28459-1-AP, 1:1000), RUNX 2 (CST, 8486, 1:1000), IBSP (Invitrogen, PA5-114915, 1:1000), Phospho-PKM2 (Invitrogen, PA5-37684, 1:1000), Histone H3 (CST, 4499, 1:2000), GAPDH (CST, 5174, 1:1000), Vinculin (CST, 13901, 1:1000) and β-Actin (CST, 4970, 1:1000). After incubation with the primary antibodies, the membranes were washed three times with TBST and incubated with the appropriate horseradish peroxidase-conjugated secondary antibodies (absin, 20002, 1:5000) for 2 h at room temperature. After another three TBST washes, luminescence reagent (Sparkjade ECL super, ED0015, Shandong Sparkjade Biotechnology Co., Ltd.) was applied, and the target protein bands were visualized via ChampChemi® 610 Plus Imaging. Band intensities were analyzed via ImageJ software, and relative protein expression levels were normalized to those of GAPDH, Vinculin, β-Actin, or Histone H3, depending on the subcellular localization of the target proteins.

### Plasmid construction and transfection

Expression plasmid constructs, including pcDNA3.1-Flag-GIPC2, pcDNA3.1-Flag-GIPC2-ΔPDZ (PDZ domain deletion), pcDNA3.1-Flag-GIPC2-ΔGH1 (GH1 domain deletion), pcDNA3.1-Flag-GIPC2-ΔGH2 (GH2 domain deletion), pcDNA3.1-HA-PKM2-ΔN + A1 (N + A1 domain deletion), pcDNA3.1-HA-PKM2-ΔB (B domain deletion), pcDNA3.1-HA-PKM2-ΔA2 (A2 domain deletion), pcDNA3.1-HA-PKM2-ΔC (C domain deletion), and pcDNA3.1-HA-PKM2 (full-length), were designed and synthesized by GeneChem (Shanghai). Transfection was performed using Lipofectamine 3000 (Invitrogen, L3000-015) following the manufacturer’s instructions. Briefly, 293T cells were seeded in 6-well plates at a density of 3 × 10⁵ cells/well in high-glucose DMEM (Procell, PM150210) supplemented with 10% FBS and incubated overnight at 37 °C with 5% CO₂ until 70–80% confluency was achieved. For each transfection, 2.5 µg of plasmid DNA (pcDNA3.1-Flag-GIPC2, deletion mutants, or empty pcDNA3.1 vector balanced for total DNA) was diluted in 125 µL Opti-MEM, mixed with 5 µL P3000 reagent, and combined with 5 µL Lipofectamine 3000 reagent (pre-diluted in an equal volume of Opti-MEM). The mixture was incubated at room temperature for 10–15 min to form DNA-lipid complexes, which were added dropwise to the cells. Transfected cells were maintained at 37 °C with 5% CO₂ for 24–48 h. Transfection efficiency was validated by WB analysis or fluorescence-based detection of tagged PKM2 or GIPC2 constructs. Cells were subsequently harvested for downstream analyses, including co-immunoprecipitation (Co-IP) and WB, to assess GIPC2 and PKM2 functionality and domain-specific activities.

### Lentiviral construction and infection

Lentiviruses for GIPC2 overexpression and knockdown (OE/KD-GIPC2) with vector controls (CON), PKM2 overexpression and knockdown (OE/KD-PKM2) with vector controls (CON), and SREBP1 knockdown (KD-SREBP1) with vector controls (CON) were purchased from OBIO Co., Ltd. (Shanghai). Lentiviral particles were produced by co-transfecting 293T cells with the lentiviral vector CV572/mCherry (OBIO) and packaging plasmids (pGag/Pol/Rev and pVSV-G) using the Lipofectamine 3000 transfection system. After 72 h, viral supernatants were collected, filtered through 0.45 µm membranes to remove cellular debris, and concentrated by ultracentrifugation. Viral titers were adjusted to 10⁸ TU/mL and mixed with MSC culture medium. MSCs were co-cultured with lentivirus-containing medium for 24 h to ensure efficient transduction. Transduced cells were selected with puromycin, and >90% infection efficiency was confirmed by mCherry fluorescence. These lentiviral-transduced MSCs were subsequently used to evaluate the functional role of modulating the GIPC2-PKM2-SREBP1 axis in adipogenic differentiation.

### Cell proliferation experiment

Cell proliferation was assessed via an EdU Cell Proliferation Assay Kit (Beyotime, C0071S) according to the manufacturer’s instructions. MSCs were seeded onto 15 mm glass-bottom cell culture dishes (SAINING Biotechnology, 1050001) at a density of 3 × 10⁵ cells per dish and incubated with 2 mL of 10 μM EdU reagent for 24 h at 37 °C with 5% CO₂. After incubation, the cells were fixed with 4% paraformaldehyde for 15 min at room temperature and washed three times with PBS (pH 7.4) for 5 min each. Permeabilization was performed using 0.3% Triton ×−100 at room temperature for 8 min, followed by three additional PBS washes. The EdU detection reagent was then added, and the cells were incubated in the dark at room temperature for 30 min. The cell nuclei were then stained with Hoechst (Beyotime, 33342) for 10 min to visualize total cell counts. The stained cells were observed via laser scanning confocal microscopy, and the fluorescence signals for EdU and Hoechst were analyzed via ImageJ software to calculate the percentage of EdU-positive cells, which reflects the proportion of newly added cell populations.

### Immunofluorescence staining

In immunofluorescence experiments, the PBS-washed cells were fixed with 4% paraformaldehyde at room temperature for 15 min to preserve the cellular structures and proteins, followed by three 5-min washes with PBS (pH 7.4). Permeabilization was performed with 0.3% Triton ×−100 for 8 min at room temperature, followed by three additional 5-min washes with PBS. For blocking of nonspecific binding, the cells were incubated with immunostaining blocking buffer at room temperature for at least 30 min. The cells were subsequently incubated overnight at 4 °C with the specific primary antibodies diluted in blocking buffer: GIPC2 (Abcam, ab175272, 1:100), PKM2 (Cell Signaling Technology, CST, 46687S, 1:100), SREBP1(Abcam, ab28481, 1:200). After overnight incubation, the cells were washed three times with PBS for 5 min each, followed by incubation with fluorescently labeled secondary antibodies at room temperature for 2 h in the dark. The cells were then washed three times with PBS, and the cell nuclei were counterstained with DAPI for 5 min to visualize the nuclear structures. Finally, the stained samples were imaged via a laser scanning confocal microscope. Fluorescent signals for GIPC2-, PKM2-, SREBP1-, and DAPI-stained nuclei were captured via laser microscope at appropriate excitation and emission wavelengths, and the images were analyzed via ImageJ software to quantify the fluorescence intensity.

### Co-IP

In subsequent protein interaction experiments, all the cells were lysed and co-immunoprecipitated with the Flag-tag Protein IP Assay Kit with Agarose Gel (Beyotime, P2202M) or HA-tag Protein IP Assay Kit with Agarose Gel (Beyotime, P2206M). The primary antibodies used in this study included GIPC2 (Abcam, ab175272, 1:1000) and PKM2 (CST, 46687S, 1:1000). The immunoprecipitate was collected, washed, and boiled in Laemmli sample buffer for 10 min. Immunoblotting was performed on the immunoprecipitated samples to detect coprecipitated proteins. Total lysate was used as the starting control for WB analysis. Protein interactions were quantified via ImageJ software.

### SREBP1 inhibitor preparation and MSC treatment

For evaluation of the role of SREBP1 in PKM2-mediated adipogenesis, the SREBP1 inhibitor Fatostatin (MedChemExpress; HY-14452) was prepared at a final concentration of 20 μM by dissolving it in lipid differentiation medium according to the manufacturer’s instructions. MSCs were seeded into 6-well plates at a density of 3 × 10⁵ cells per well, and once the cells reached the appropriate confluency, adipogenic differentiation was induced via lipid differentiation medium containing 20 μM Fatostatin. The control group was treated with the same adipogenic differentiation medium but without Fatostatin. The cells were maintained in a humidified incubator at 37 °C with 5% CO₂, and the differentiation medium (with or without Fatostatin) was changed every 2–3 days. On the 15th day of differentiation, the cells were harvested for further analysis.

### Separation of the nuclear cytoplasm

Nuclear and cytoplasmic proteins from cells were separated via the Thermo Scientific NE-PER Nuclear and Cytoplasmic Extraction Kit (catalog No. 78833) according to the manufacturer’s protocol. Briefly, cells were harvested at the specified time points and washed twice with cold PBS (pH 7.4). The cell pellet was then resuspended in Cytoplasmic Extraction Reagent I (CER I), vortexed vigorously for 15 s, and incubated on ice for 10 min. Cytoplasmic Extraction Reagent II (CER II) was subsequently added, followed by another 5 s of vortexing and a 1-min incubation on ice. The mixture was centrifuged at 16,000 × *g* for 5 min at 4 °C, and the supernatant containing the cytoplasmic proteins was carefully collected. The remaining pellet was resuspended in Nuclear Extraction Reagent and vortexed for 15 s every 10 min for a total of 40 min on ice. The sample was then centrifuged at 16,000 × *g* for 10 min at 4 °C, and the supernatant containing the nuclear proteins was collected.

### PK activity assay

For pyruvate kinase (PK) activity measurement in 3D adipospheres or UC-MSCs with GIPC2 knockdown or overexpression, adipospheres or UC-MSCs were homogenized on ice and thoroughly lysed. Lysates were then subjected to PK enzymatic activity determination using a Pyruvate Kinase Activity Assay Kit (Solarbio, BC0454) following the manufacturer’s standard protocol. PK activity values were normalized to protein concentration.

### 3D adiposphere ATP level measurement

ATP levels in 3D adipospheres with GIPC2 knockdown or overexpression were quantified using an ATP Assay Kit (Beyotime, S0027) according to the manufacturer’s instructions. Values were normalized to those in vector control-treated adipospheres, which were set as 100%.

### Statistical analysis

All the statistical analyses were performed via GraphPad Prism software (version 9.0, GraphPad Software, San Diego, CA, USA, www.graphpad.com), and all the data are expressed as the means ± standard errors (SEMs). Independent samples *t*-tests were used to analyze differences in normally distributed data between two groups. One-way analysis of variance with Tukey’s multiple comparisons test was used to analyze differences among multiple groups. Repeated measures analysis of variance with Tukey’s multiple comparisons test was used to observe the characteristics of multiple groups at different time points. All the data were collected from at least three independent experiments. Unless otherwise specified, *n* represents the number of individual biological replicates and is represented in graphs as one dot per sample.

### Ethical approval and consent to participate

This study has been approved by the Ethics Committee of the First Affiliated Hospital of Dalian Medical University (approval number: YJ-GXB-2022-01). All procedures were conducted in accordance with the relevant guidelines and regulations.

## Supplementary information

Supplemetary data

original western blot

## Data Availability

The authors declare that all data supporting the findings of this study are available within the article.
