# Supplementary material for: GIPC2 regulation of the PKM2/SREBP1 signaling axis controls adipogenic differentiation of mesenchymal stem cells
Source: Cell Death Dis. 2026 Jan 7;17(1):5. doi: 10.1038/s41419-025-08088-9 (PMC12779644; doi:10.1038/s41419-025-08088-9)
Supplement: Supplementary file 1 — Supplemetary data [file 41419_2025_8088_MOESM1_ESM.pdf]

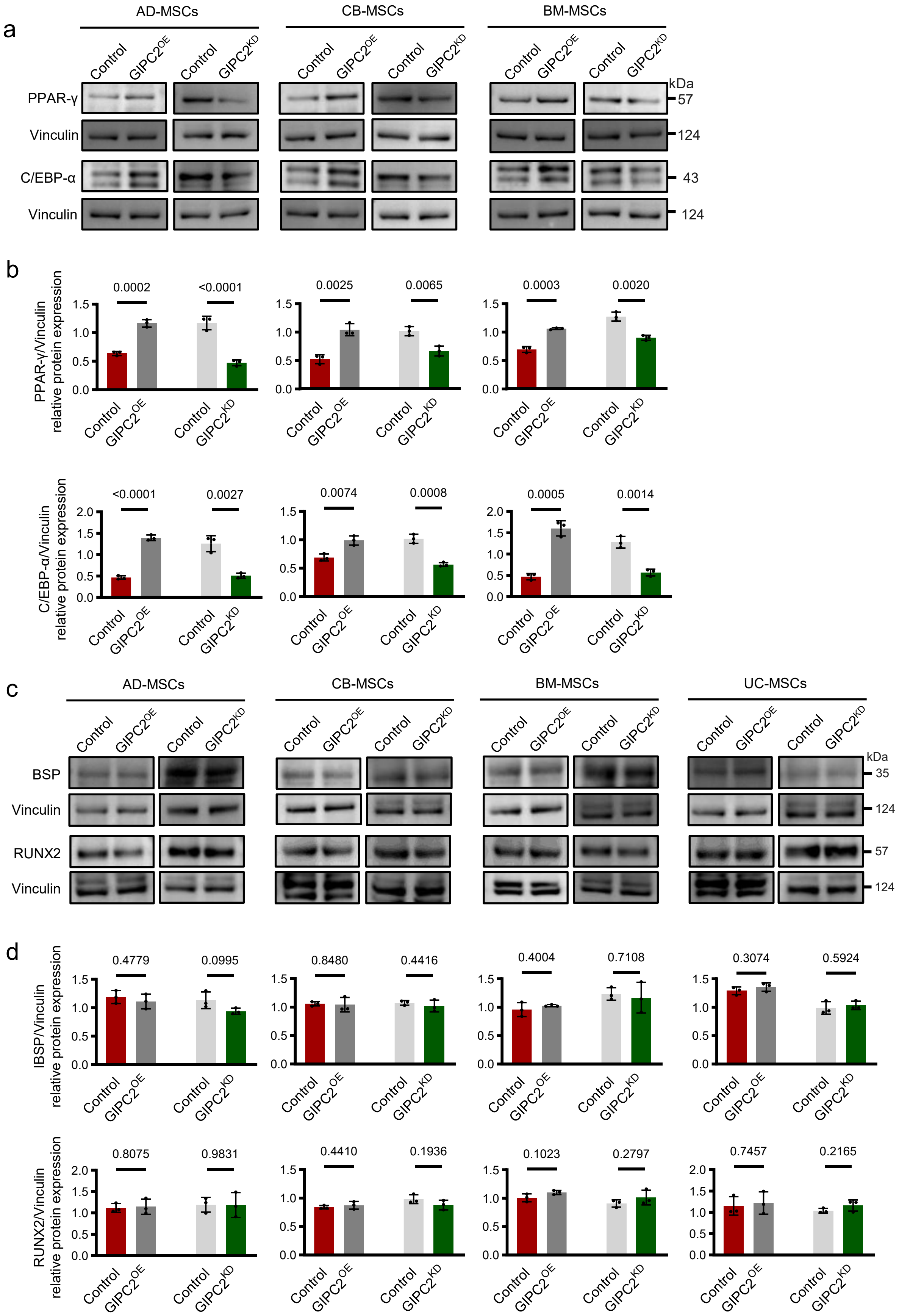

a
AD-MSCs
CB-MSCs
BM-MSCs
Control
GIPC2OE
Control
GIPC2KD
PPAR-γ
Vinculin
C/EBP-α
Vinculin
kDa
57
124
43
124
b
PPAR-γ/Vinculin relative protein expression
0.0002
<0.0001
0.0025
0.0065
0.0003
0.0020
C/EBP-α/Vinculin relative protein expression
<0.0001
0.0027
0.0074
0.0008
0.0005
0.0014
c
AD-MSCs
CB-MSCs
BM-MSCs
UC-MSCs
BSP
Vinculin
RUNX2
Vinculin
kDa
35
124
57
124
d
IBSP/Vinculin relative protein expression
0.4779
0.0995
0.8480
0.4416
0.4004
0.7108
0.3074
0.5924
RUNX2/Vinculin relative protein expression
0.8075
0.9831
0.4410
0.1936
0.1023
0.2797
0.7457
0.2165

Supplementary Figure 1. Western blot analysis of the indirect effects of GIPC2 modulation on adipogenic and osteogenic differentiation in MSCs.

(a) Western blot (WB) analysis of the adipogenic markers PPAR-γ, C/EBP-α in GIPC2-overexpressing or knockdown MSCs (AD-MSCs, CB-MSCs, BM-MSCs). (b) Quantitative analysis of adipogenic differentiation markers using WB. (c) WB analysis of the osteogenic markers IBSP, RUNX2 in GIPC2-overexpressing or knockdown MSCs (AD-MSCs, CB-MSCs, BM-MSCs, UC-MSCs). (d) Quantitative analysis of expression of osteogenic differentiation markers obtained using WB. Statistical analysis: two-sided t test (b, d). Error bars: mean ± s.e.m.

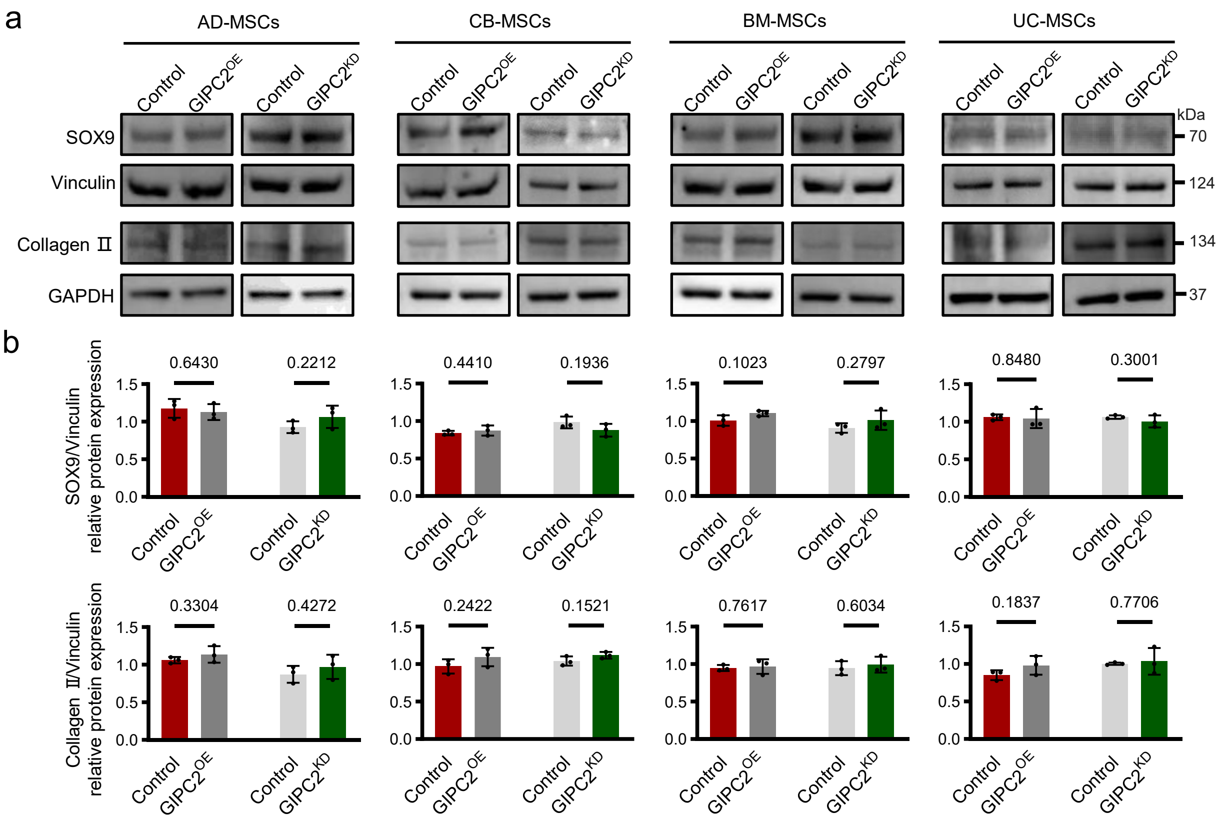

Supplementary Figure 2. WB analysis of the indirect effects of GIPC2 modulation on chondrogenic differentiation in MSCs.

(a) WB analysis of the chondrogenic markers SOX9, Collagen II in GIPC2- overexpression or knockdown MSCs (AD-MSCs, CB-MSCs, BM-MSCs, UC-MSCs). (b) Quantitative analysis of expression of chondrogenic differentiation markers obtained using WB. Statistical analysis: two-sided t test (b). Error bars: mean ± s.e.m.

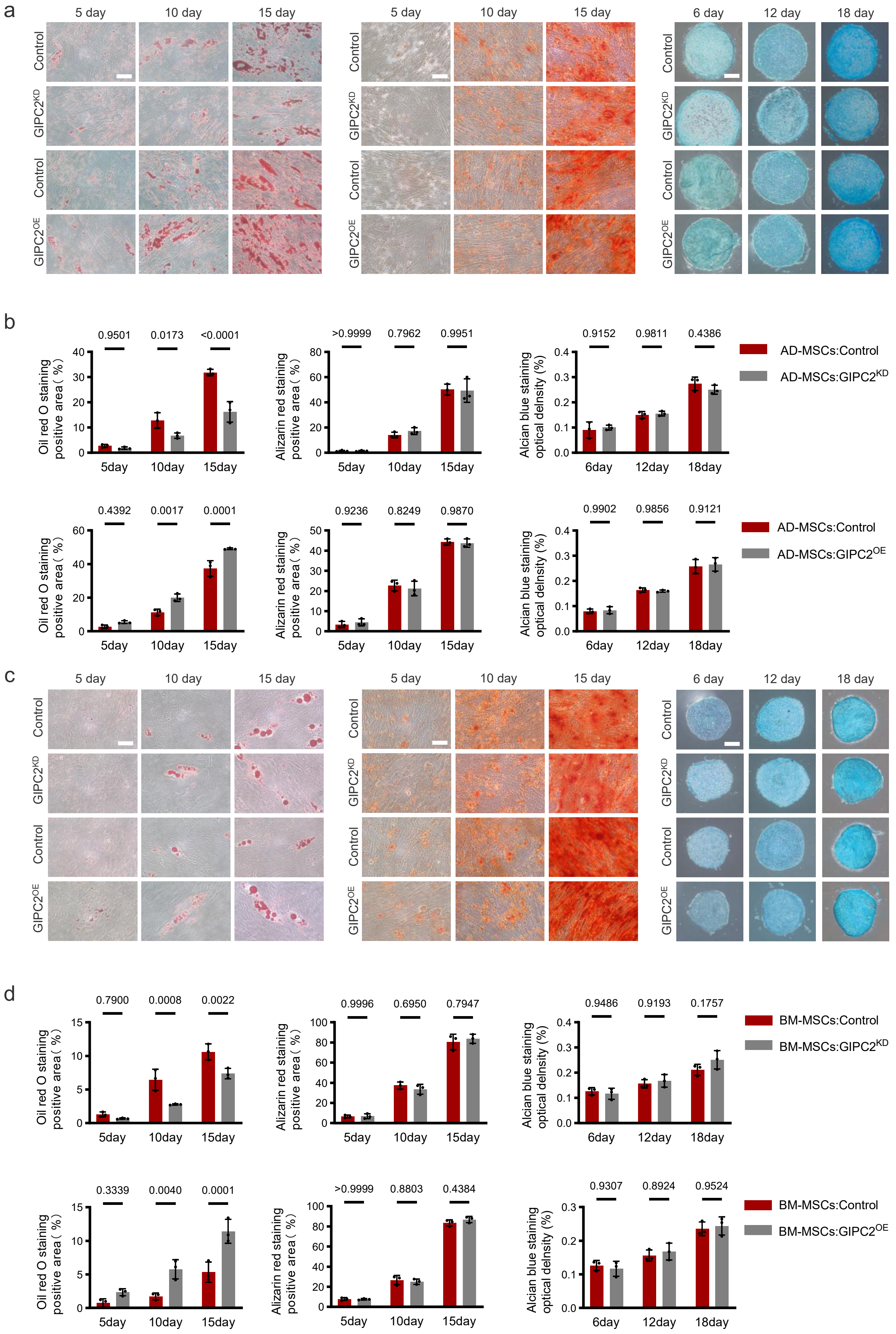
a
5 day
10 day
15 day
6 day
12 day
18 day
Control
GIPC2KD
Control
GIPC2OE
b
0.9501
0.0173
<0.0001
>0.9999
0.7962
0.9951
0.9152
0.9811
0.4386
Oil red O staining positive area (%)
Alizarin red staining positive area (%)
Alcian blue staining optical delnsity (%)
5day
10day
15day
6day
12day
18day
AD-MSCs:Control
AD-MSCs:GIPC2KD
0.4392
0.0017
0.0001
0.9236
0.8249
0.9870
0.9902
0.9856
0.9121
AD-MSCs:Control
AD-MSCs:GIPC2OE
c
d
0.7900
0.0008
0.0022
0.9996
0.6950
0.7947
0.9486
0.9193
0.1757
BM-MSCs:Control
BM-MSCs:GIPC2KD
0.3339
0.0040
0.0001
>0.9999
0.8803
0.4384
0.9307
0.8924
0.9524
BM-MSCs:Control
BM-MSCs:GIPC2OE

Supplementary Figure 3. Trilineage staining assessment of GIPC2 modulation effects on differentiation in MSCs.

(a) ORO staining, Alizarin Red staining and Alcian Blue Staining at multiple time points during adipogenic induction of AD-MSCs. (b) Quantitative analysis chart of ORO, Alizarin Red, and Alcian Blue staining in AD-MSCs. (c) ORO staining, Alizarin Red staining and Alcian Blue Staining at multiple time points during adipogenic induction of BM-MSCs. (d) Quantitative analysis chart of ORO, Alizarin Red, and Alcian Blue staining in BM-MSCs. Statistical analysis: two-sided t test (b,d). Error bars: mean ± s.e.m. Scale bar, 50 μm (a,c).

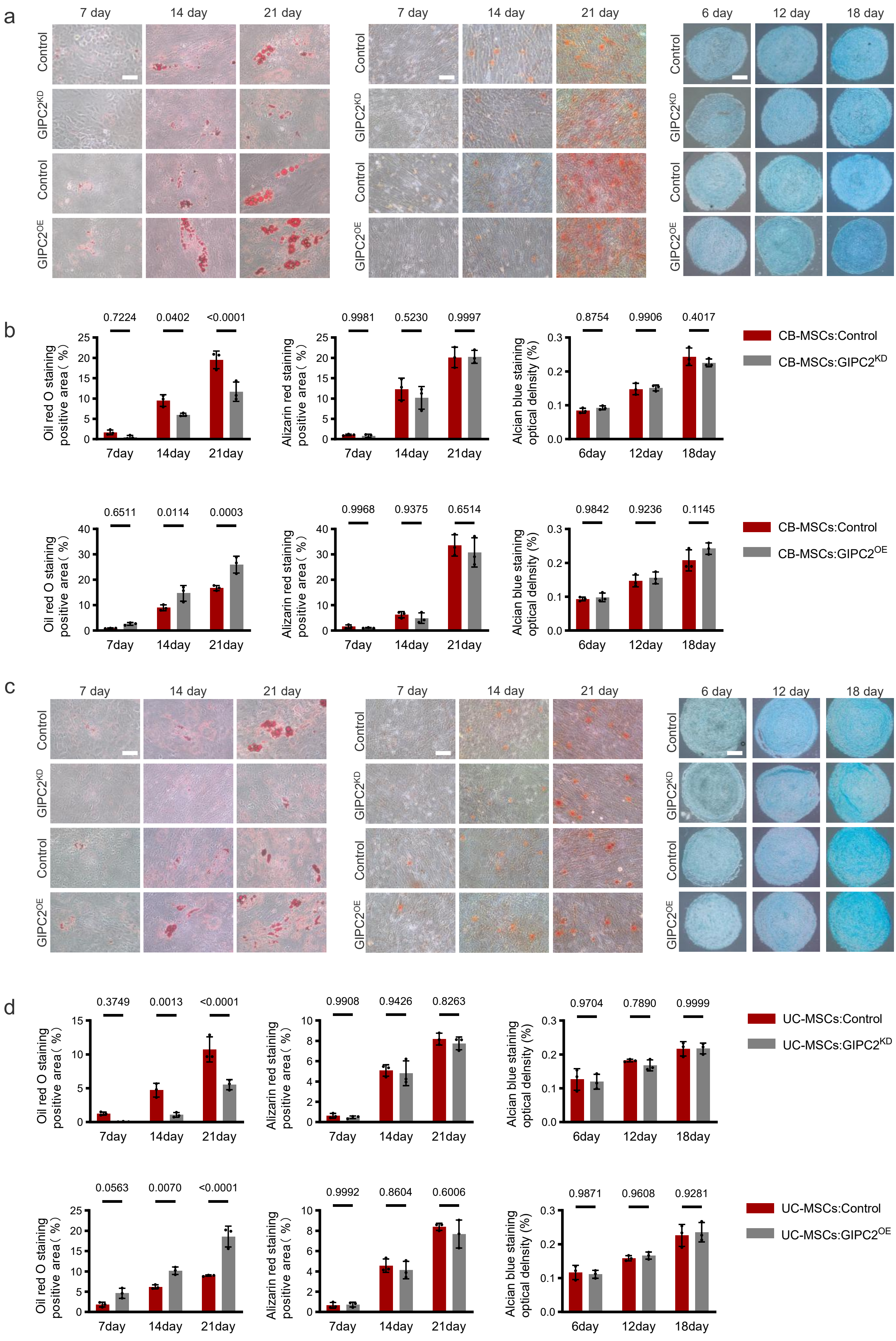

a
7 day
14 day
21 day
6 day
12 day
18 day
Control
GIPC2KD
GIPC2OE
b
0.7224
0.0402
<0.0001
0.9981
0.5230
0.9997
0.8754
0.9906
0.4017
Oil red O staining positive area (%)
Alizarin red staining positive area (%)
Alcian blue staining optical delnsity (%)
7day
14day
21day
6day
12day
18day
CB-MSCs:Control
CB-MSCs:GIPC2KD
0.6511
0.0114
0.0003
0.9968
0.9375
0.6514
0.9842
0.9236
0.1145
CB-MSCs:GIPC2OE
c
d
0.3749
0.0013
<0.0001
0.9908
0.9426
0.8263
0.9704
0.7890
0.9999
UC-MSCs:Control
UC-MSCs:GIPC2KD
0.0563
0.0070
<0.0001
0.9992
0.8604
0.6006
0.9871
0.9608
0.9281
UC-MSCs:GIPC2OE

Supplementary Figure 4. Trilineage staining assessment of GIPC2 modulation effects on differentiation in MSCs.

(a) ORO staining, Alizarin Red staining and Alcian Blue Staining at multiple time points during adipogenic induction of CB-MSCs. (b) Quantitative analysis chart of ORO, Alizarin Red, and Alcian Blue staining in CB-MSCs. (c) ORO staining, Alizarin Red staining and Alcian Blue Staining at multiple time points during adipogenic induction of UC-MSCs. (d) Quantitative analysis chart of ORO, Alizarin Red, and Alcian Blue staining in UC-MSCs. Statistical analysis: two-sided t test (b,d). Error bars: mean ± s.e.m. Scale bar, 50 μm (a,c).

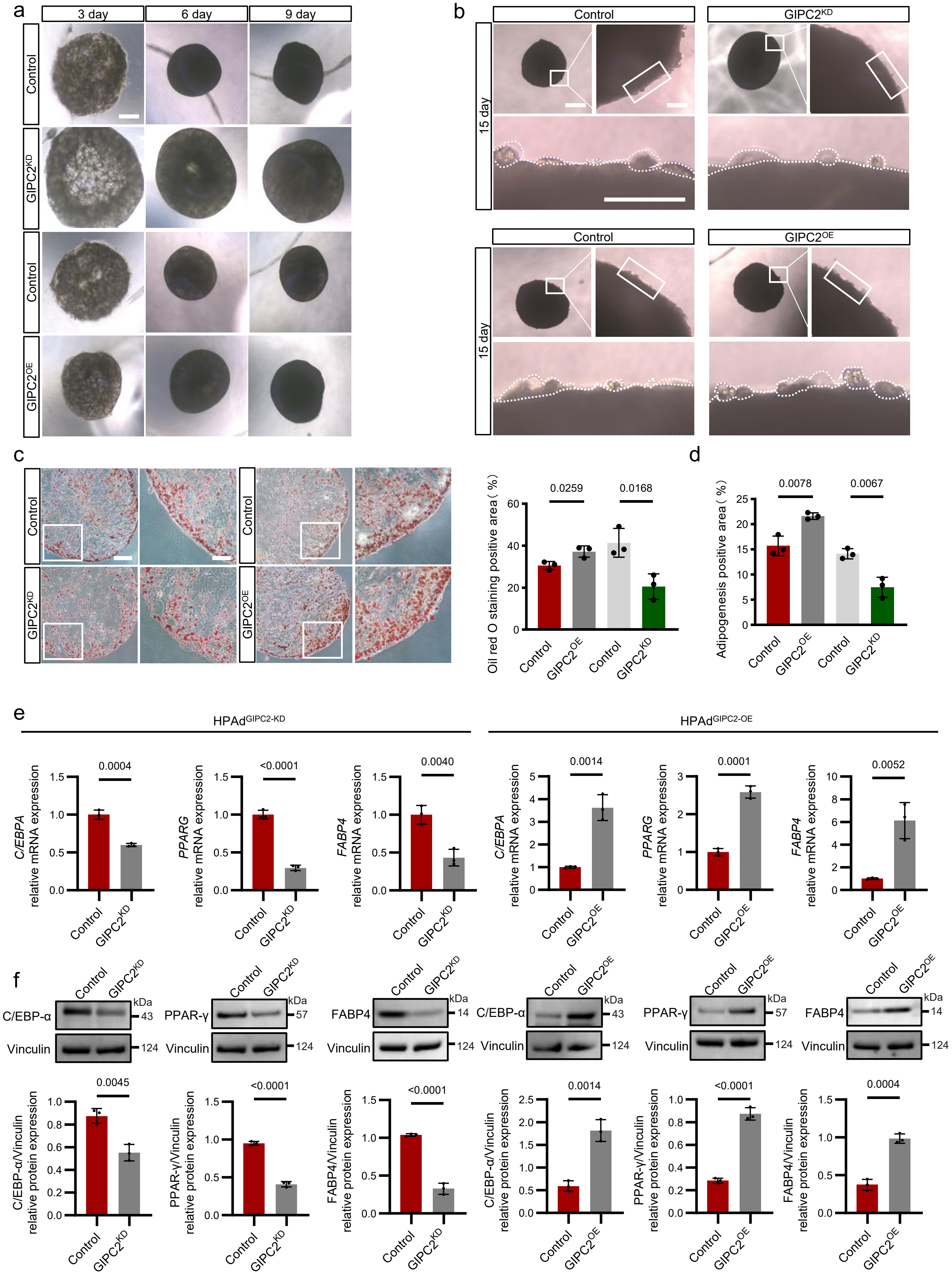

a
3 day
6 day
9 day
Control
GIPC2KD
Control
GIPC2OE
b
Control
GIPC2KD
15 day
Control
GIPC2OE
15 day
c
Control
GIPC2KD
Control
GIPC2OE
Oil red O staining positive area (%)
0.0259
0.0168
Control
GIPC2OE
Control
GIPC2KD
d
Adipogenesis positive area (%)
0.0078
0.0067
Control
GIPC2OE
Control
GIPC2KD
e
HPAdGIPC2-KD
HPAdGIPC2-OE
C/EBPA relative mRNA expression
0.0004
PPARG relative mRNA expression
<0.0001
FABP4 relative mRNA expression
0.0040
C/EBPA relative mRNA expression
0.0014
PPARG relative mRNA expression
0.0001
FABP4 relative mRNA expression
0.0052
Control
GIPC2KD
Control
GIPC2OE
f
C/EBP-α
PPAR-γ
FABP4
Vinculin
kDa
43
57
14
124
C/EBP-α/Vinculin relative protein expression
0.0045
PPAR-γ/Vinculin relative protein expression
<0.0001
FABP4/Vinculin relative protein expression
<0.0001
C/EBP-α/Vinculin relative protein expression
0.0014
PPAR-γ/Vinculin relative protein expression
<0.0001
FABP4/Vinculin relative protein expression
0.0004
Control
GIPC2KD
Control
GIPC2OE

Supplementary Figure 5. GIPC2 regulates the assembly of 3D adipospheres and adipogenic capacity.

(a) Optical microscopy monitoring of GIPC2 regulatory effects on 3D adiposphere growth under different days of growth. (b) Quantitative analysis of protruding lipid droplets on adiposphere surfaces on day 15 of adipogenic differentiation. (c) Cryosectioning and ORO staining analysis of differentiated 3D adipospheres. (d) Quantitative analysis chart of (b). (e) qRT-PCR analysis to assess the effects of GIPC2 overexpression or knockdown on key adipogenic genes (*PPARG, C/EBPA*, and *FABP4*) in 3D adipospheres. (f) WB analysis to assess the effects of GIPC2 overexpression or knockdown on key adipogenic marker proteins (PPAR-γ, C/EBP-α, and FABP4) in 3D adipospheres. Statistical analysis: two-sided t test (c–f). Error bars: mean ± s.e.m. Scale bar, 150 μm (a,b), 100 μm (c), 50 μm (b,c enlarged image).

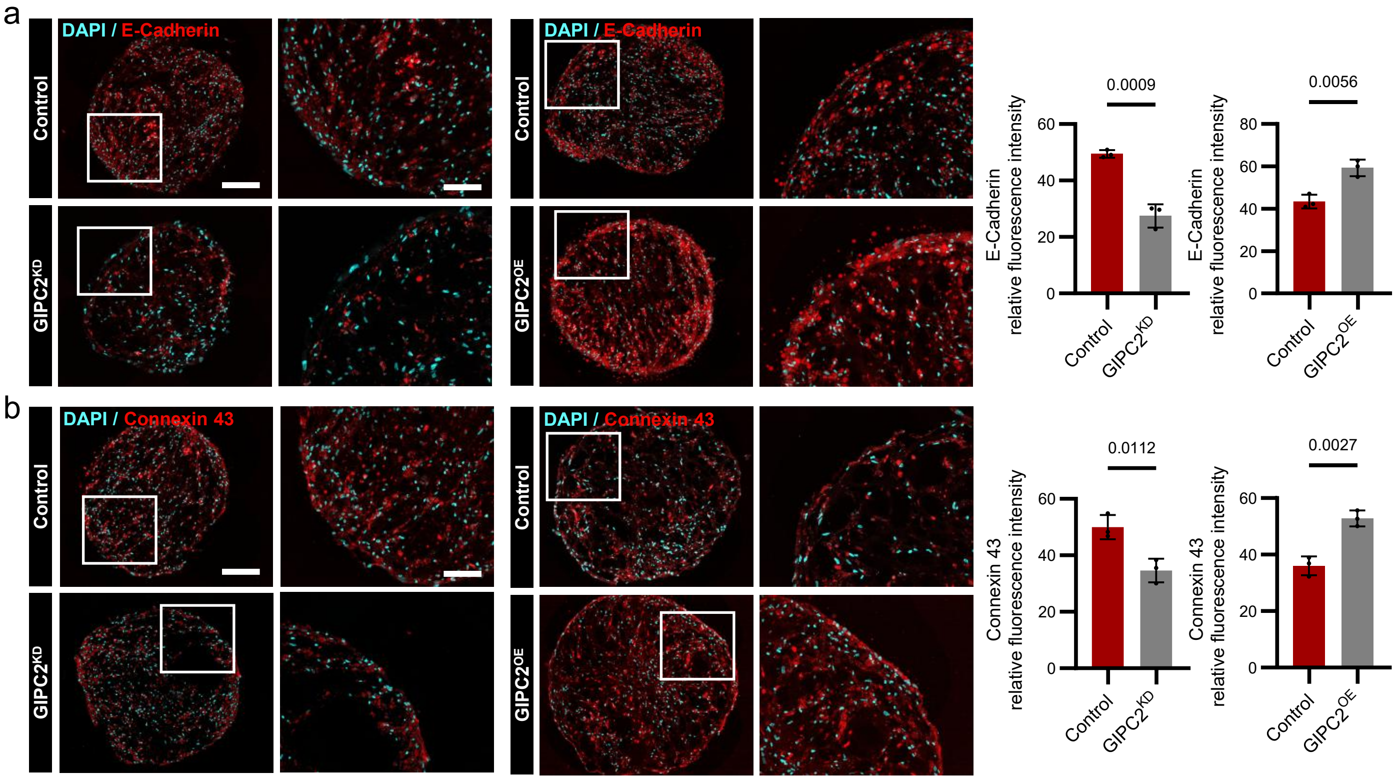

Supplementary Figure 6. GIPC2 regulates cell junctions in 3D adipospheres.

(a) Cryosectioning followed by E-cadherin immunofluorescence detection and quantitative analysis in differentiated 3D adipospheres. (b) Cryosectioning followed by connexin 43 immunofluorescence detection and quantitative analysis in differentiated 3D adipospheres. Statistical analysis: two-sided t test (a,b). Error bars: mean ± s.e.m. Scale bar, 100 µm (a,b), 50 µm (a,b enlarged image).

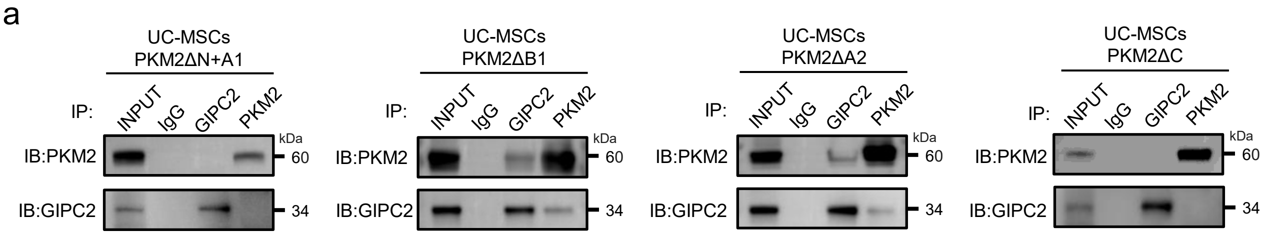

Supplementary Figure 7. (a) Co-immunoprecipitation (Co-IP) of GIPC2 with PKM2 carrying domain-specific mutations.

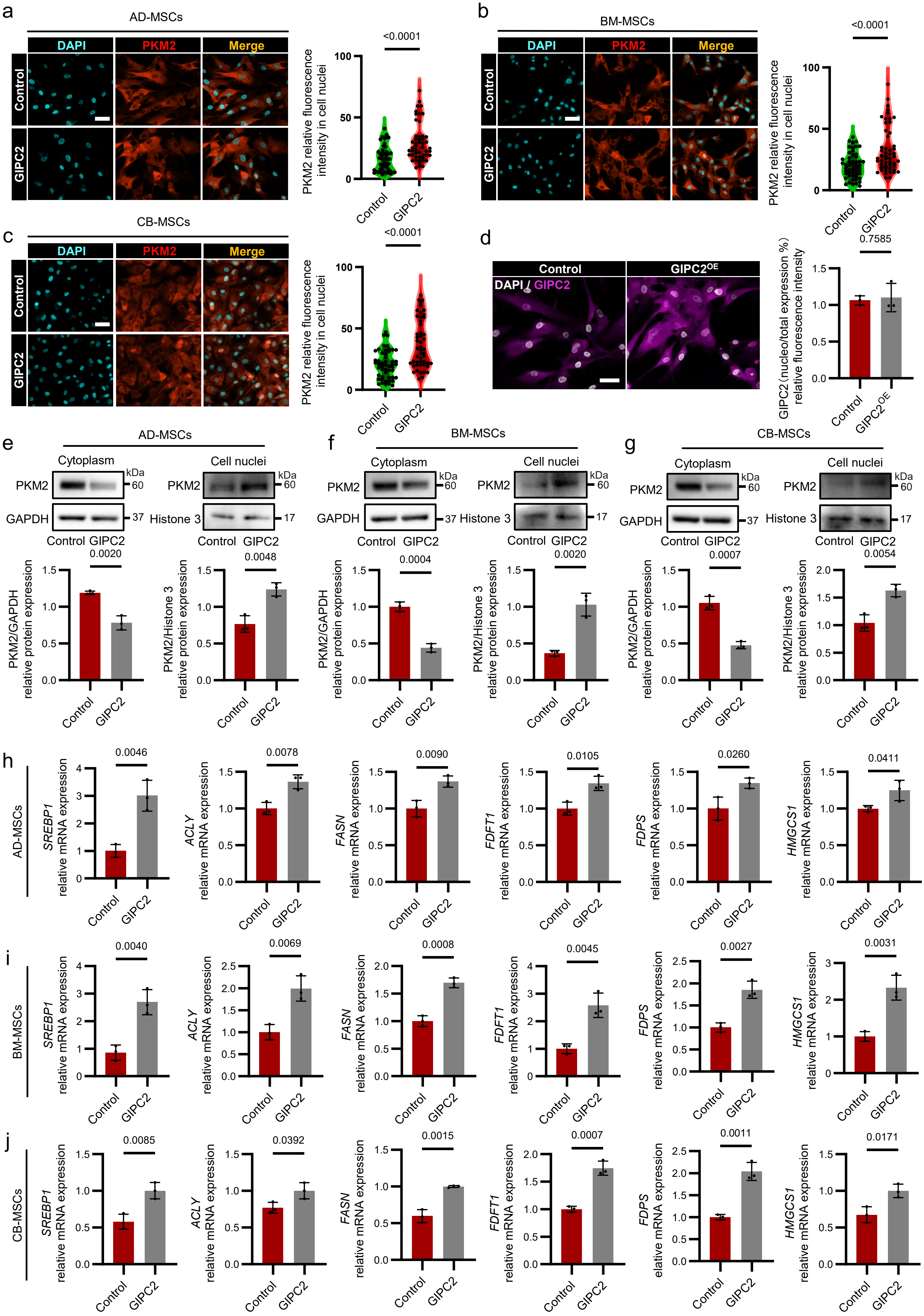

a
AD-MSCs
DAPI
PKM2
Merge
Control
GIPC2
PKM2 relative fluorescence intensity in cell nuclei
<0.0001
b
BM-MSCs
DAPI
PKM2
Merge
Control
GIPC2
PKM2 relative fluorescence intensity in cell nuclei
<0.0001
c
CB-MSCs
DAPI
PKM2
Merge
Control
GIPC2
PKM2 relative fluorescence intensity in cell nuclei
<0.0001
d
Control
GIPC2^{OE}
DAPI / GIPC2
GIPC2 (nucleo/total expression %) relative fluorescence intensity
0.7585
e
AD-MSCs
Cytoplasm
Cell nuclei
PKM2
GAPDH
Histone 3
kDa
60
37
17
Control GIPC2
PKM2/GAPDH relative protein expression
0.0020
PKM2/Histone 3 relative protein expression
0.0048
f
BM-MSCs
Cytoplasm
Cell nuclei
PKM2/GAPDH relative protein expression
0.0004
PKM2/Histone 3 relative protein expression
0.0020
g
CB-MSCs
Cytoplasm
Cell nuclei
PKM2/GAPDH relative protein expression
0.0007
PKM2/Histone 3 relative protein expression
0.0054
h
AD-MSCs
SREBP1 relative mRNA expression 0.0046
ACLY relative mRNA expression 0.0078
FASN relative mRNA expression 0.0090
FDFT1 relative mRNA expression 0.0105
FDPS relative mRNA expression 0.0260
HMGCS1 relative mRNA expression 0.0411
i
BM-MSCs
SREBP1 relative mRNA expression 0.0040
ACLY relative mRNA expression 0.0069
FASN relative mRNA expression 0.0008
FDFT1 relative mRNA expression 0.0045
FDPS relative mRNA expression 0.0027
HMGCS1 relative mRNA expression 0.0031
j
CB-MSCs
SREBP1 relative mRNA expression 0.0085
ACLY relative mRNA expression 0.0392
FASN relative mRNA expression 0.0015
FDFT1 relative mRNA expression 0.0007
FDPS elative mRNA expression 0.0011
HMGCS1 relative mRNA expression 0.0171
Control
GIPC2

Supplementary Figure 8. The regulatory role of the GIPC2-PKM2-SREBP1 axis is broadly conserved in MSCs derived from diverse cell lines.

(a–c) Immunofluorescence detection of GIPC2 overexpression-promoted nuclear translocation of PKM2 in (a) AD-MSCs, (b) BM-MSCs, and (c) CB-MSCs. (d) Expression of GIPC2 evaluated using immunofluorescence staining in UC-MSCs with or without GIPC2 overexpression. (e–g) WB analysis of PKM2 subcellular distribution following GIPC2 overexpression in (e) AD-MSCs, (f) BM-MSCs, and (g) CB-MSCs. (h–j) qRT-PCR analysis of *SREBP1* and downstream target gene activation following GIPC2 overexpression in (g) AD-MSCs, (h) BM-MSCs, and (i) CB-MSCs. Statistical analysis: two-sided t test (a–j). Within each replicate, we analyzed ⩾50 randomly selected cells across multiple imaging fields (a–c).Error bars: mean ± s.e.m. Scale bar, 25 μm (a–d).

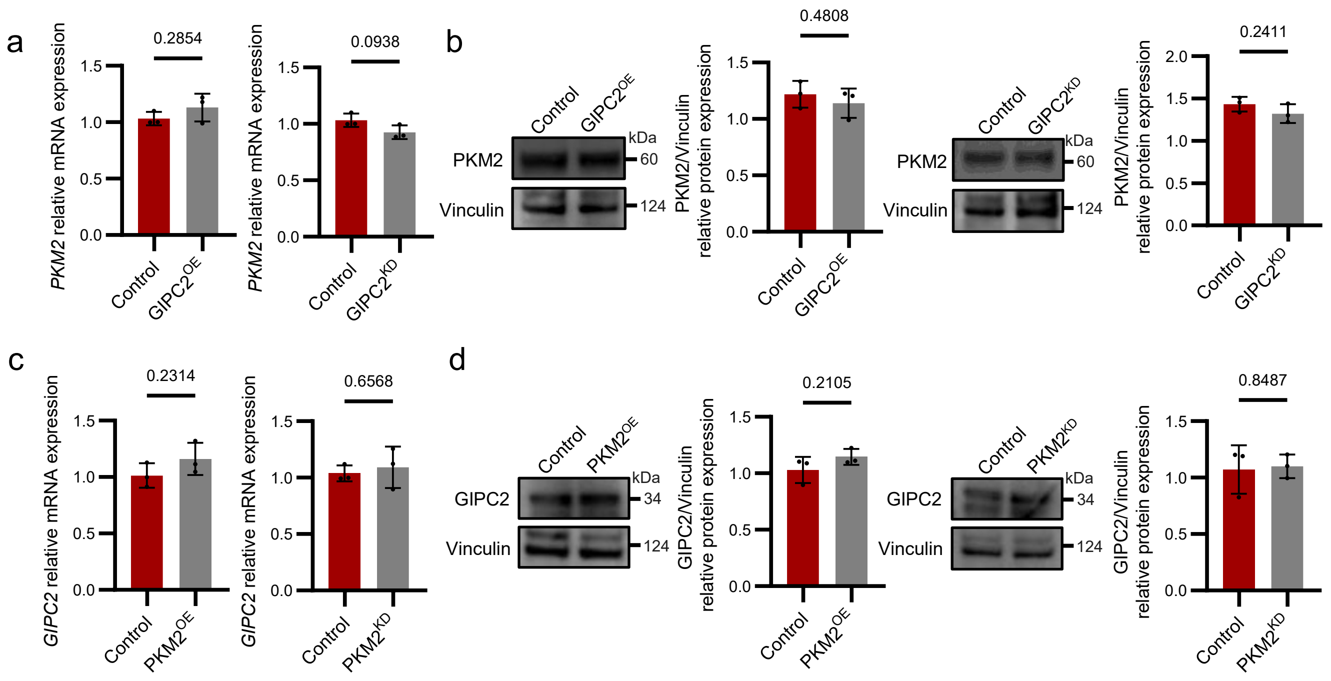

Supplementary Figure 9. GIPC2-PKM2 axis potentiates adipogenesis via unidirectional regulation of PKM2 phosphorylation.

(a, b) Transcript (a) and protein levels (b) of PKM2 in GIPC2 overexpressing or knockdown cells assessed using qRT-PCR (a) and WB (b), respectively. (c, d) Transcript (c) and protein levels (d) of GIPC2 in PKM2 overexpressing or knockdown cells assessed using qRT-PCR (c) and WB (d), respectively. Statistical analysis: two-sided t test (a–d). Error bars: mean ± s.e.m.

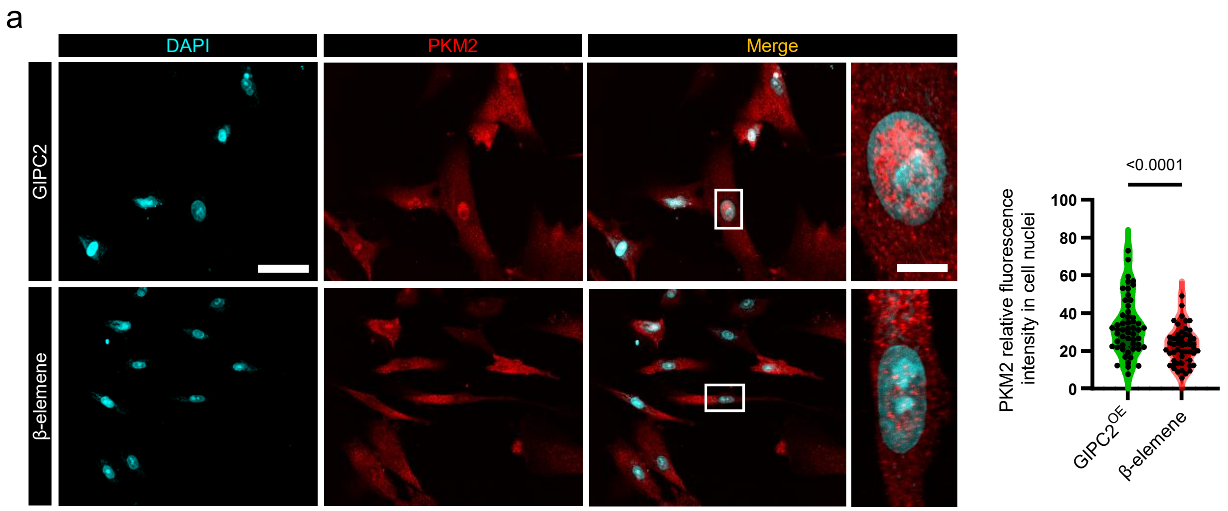

Supplementary Figure 10. (a) Immunofluorescence analyses evaluating PKM2 expression in GIPC2-overexpressing MSCs following β-elemene treatment. Statistical analysis: two-sided t test (a). Within each replicate, we analyzed ⩾50 randomly selected cells across multiple imaging fields (a). Error bars: mean ± s.e.m. Scale bar, 25 µm (a), 10 µm (a enlarged image).

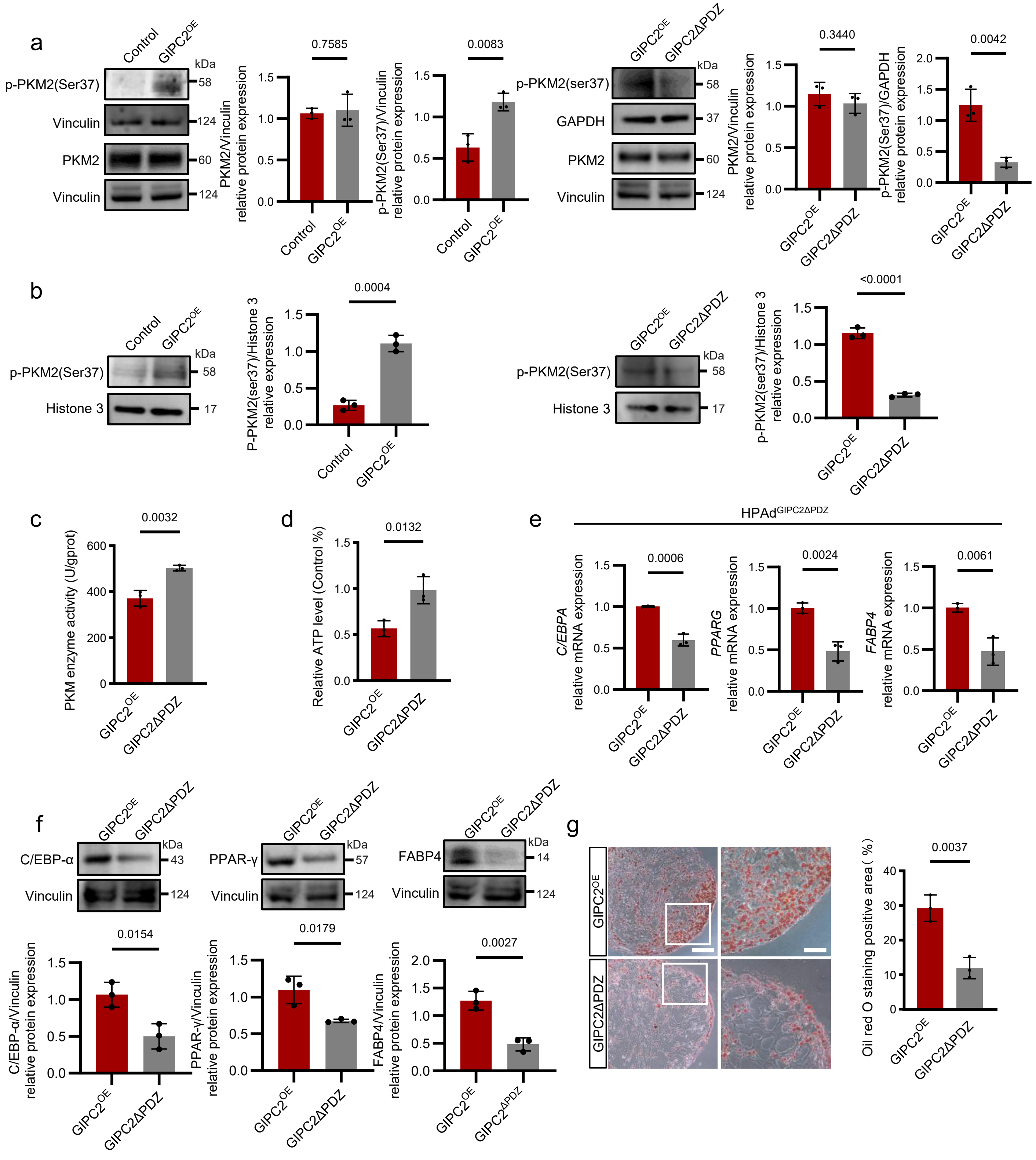

Supplementary Figure 11. GIPC2 promotes PKM2 nuclear translocation via PDZ domain-mediated phosphorylation to enhance adipogenesis.

(a, b) WB detection of PKM2 Ser37 phosphorylation levels after (a) GIPC2 overexpression and (b) mutation of the PDZ domain in GIPC2 overexpressing UC-MSCs. (c) Measurement of PKM enzymatic activity in 3D adipospheres with PDZ domain deletion. (d) Quantification of ATP production levels in 3D adipospheres following PDZ domain deletion. (e) qRT-PCR analysis of the transcript levels of adipogenic markers *PPARG*, *C/EBPA*, and *FABP4* in HPAds with either PDZ domain deletion or wild-type GIPC2. (f) WB analysis of the protein levels of the adipogenic

markers PPAR-γ, C/EBP-α, and FABP4 in HPAds with either PDZ domain deletion or wild-type GIPC2. (g) Cryosectioning and ORO staining analysis of differentiated 3D adipospheres with PDZ domain deletion or wild-type GIPC2. Statistical analysis: two-sided t test (a–g). Error bars: mean ± s.e.m. Scale bar, 100 μm (g), 50 μm (g enlarged image).

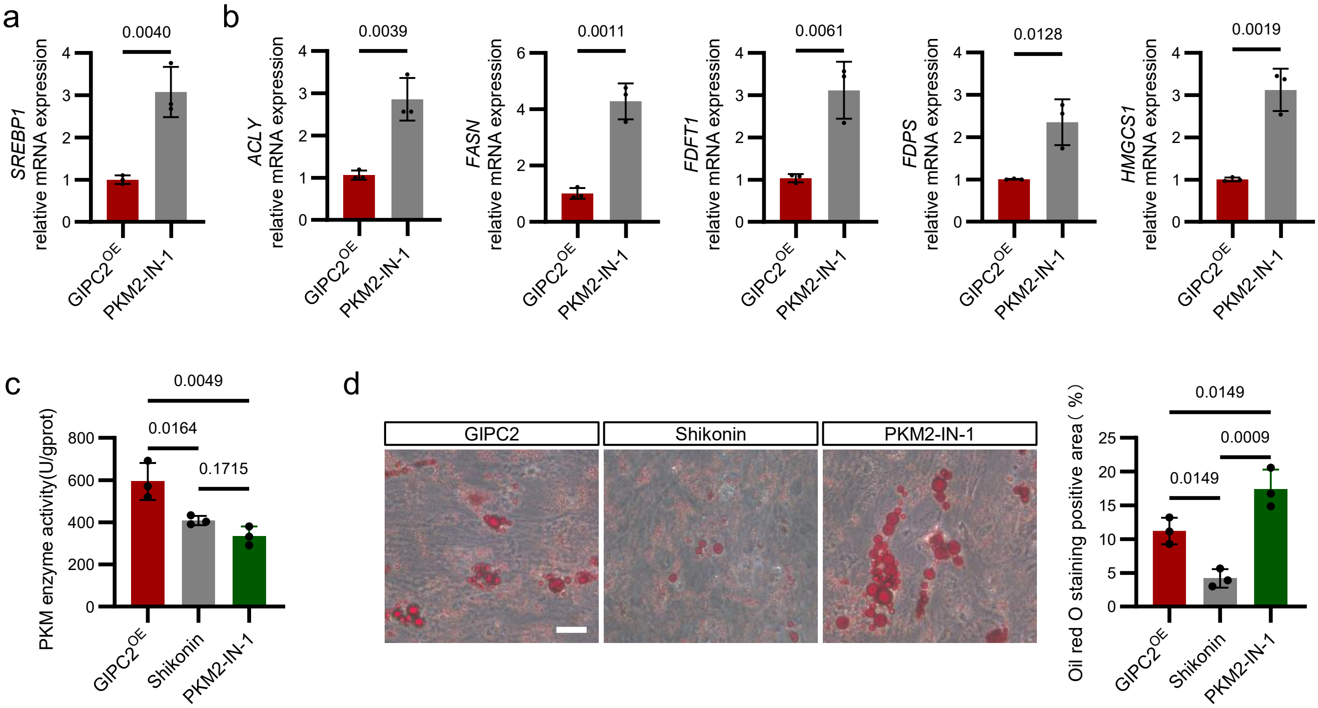

Supplementary Figure 12. The GIPC2-PKM2-SREBP1 axis regulates adipogenesis independently of PKM2 enzymatic activity.

(a) qRT-PCR analysis of *SREBP1* in GIPC2-overexpressing MSCs following PKM2-IN-1 treatment. (b) qRT-PCR analysis of *ACLY, FASN, FDPS, FDFT1*, and *HMGCS1* in GIPC2-overexpressing MSCs following PKM2-IN-1 treatment. (c) Measurement of PKM enzymatic activity in GIPC2-overexpressing MSCs treated with Shikonin or PKM2-IN-1. (d) ORO staining used to analyze lipid droplet formation in GIPC2-overexpressing MSCs treated with Shikonin or PKM2-IN-1 following 15 days of adipogenic induction. Statistical analysis: two-sided t test (a, b, d), one-way ANOVA (c). Error bars: mean ± s.e.m. Scale bar, 50 μm (d).

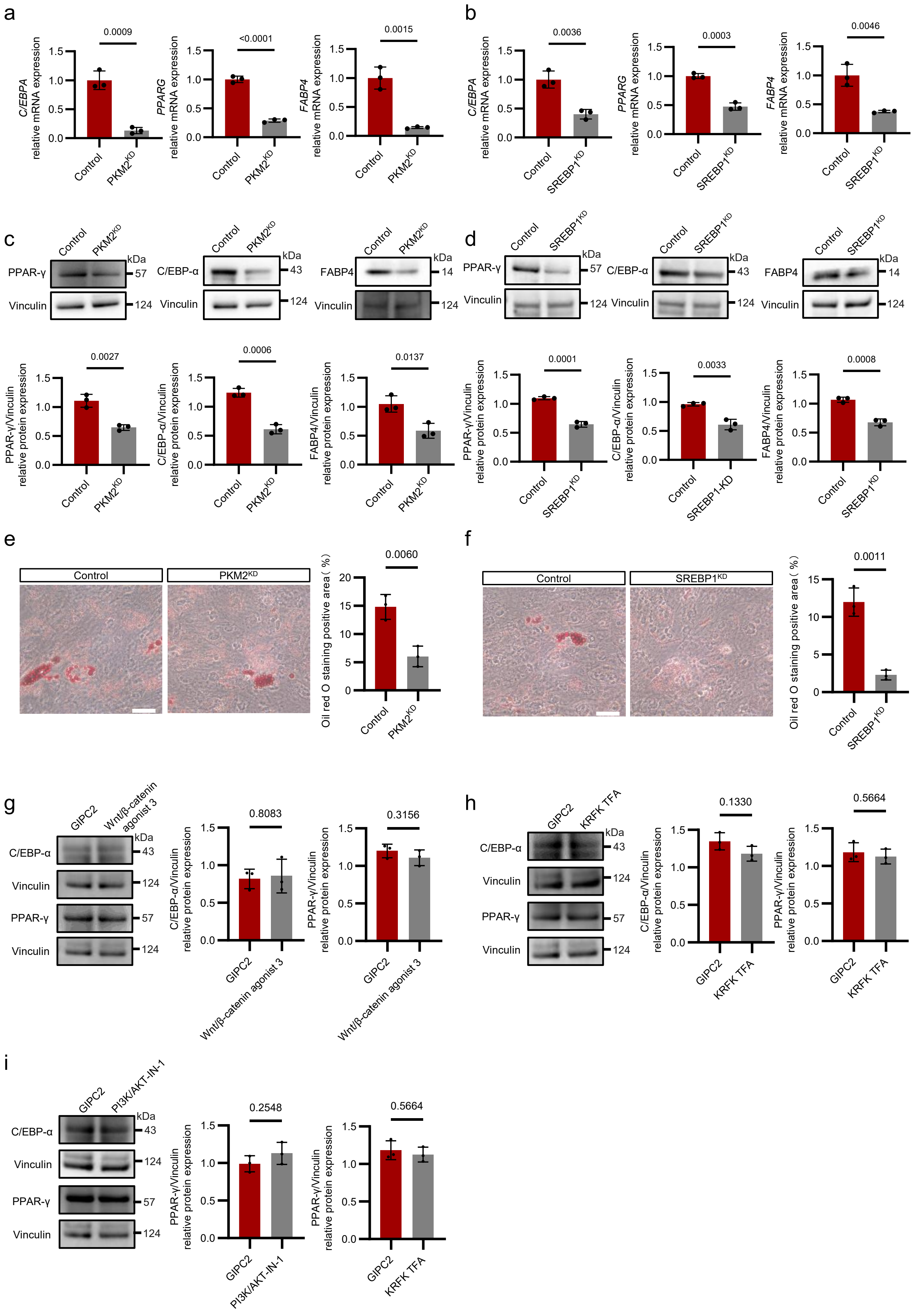

a
C/EBPA relative mRNA expression
0.0009
Control
PKM2KD
PPARG relative mRNA expression
<0.0001
FABP4 relative mRNA expression
0.0015
b
0.0036
SREBP1KD
0.0003
0.0046
c
PPAR-γ
Vinculin
C/EBP-α
FABP4
kDa
57
124
43
14
PPAR-γ/Vinculin relative protein expression
0.0027
C/EBP-α/Vinculin relative protein expression
0.0006
FABP4/Vinculin relative protein expression
0.0137
d
0.0001
0.0033
SREBP1-KD
0.0008
e
Oil red O staining positive area (%)
0.0060
f
0.0011
g
GIPC2
Wnt/β-catenin agonist 3
0.8083
0.3156
h
KRFK TFA
0.1330
0.5664
i
PI3K/AKT-IN-1
0.2548
0.5664

Supplementary Figure 13. The GIPC2-PKM2-SREBP1 axis regulates adipogenesis as an independent pathway, and the knockdown of either PKM2 or SREBP1 can block its function. (a, b) qRT-PCR used to assess the transcript levels of the adipogenic markers *PPARG, C/EBPA*, and *FABP4* in cells with (a) PKM2 and (b) SREBP1 knockdown. (c, d) WB analysis used to evaluate the protein levels of the adipogenic markers PPAR-γ, C/EBP-α, and FABP4 in cells with (c) PKM2 and (d) SREBP1 knockdown. (e, f) ORO staining used to visualize lipid droplet formation during trilineage differentiation of MSCs with (e) PKM2 and (f) SREBP1 knockdown. (g–i) WB analysis of the protein levels of the adipogenic markers PPAR-γ, C/EBP-α in GIPC2-overexpressing MSCs (g) with or without Wnt/β-catenin agonist 3 and (h) with or without KRFK TFA, and (i) with or without PI3K/AKT-IN-1 treatments. Statistical analysis: two-sided t test (a–i). Error bars: mean ± s.e.m. Scale bar, 50 µm (e, f).

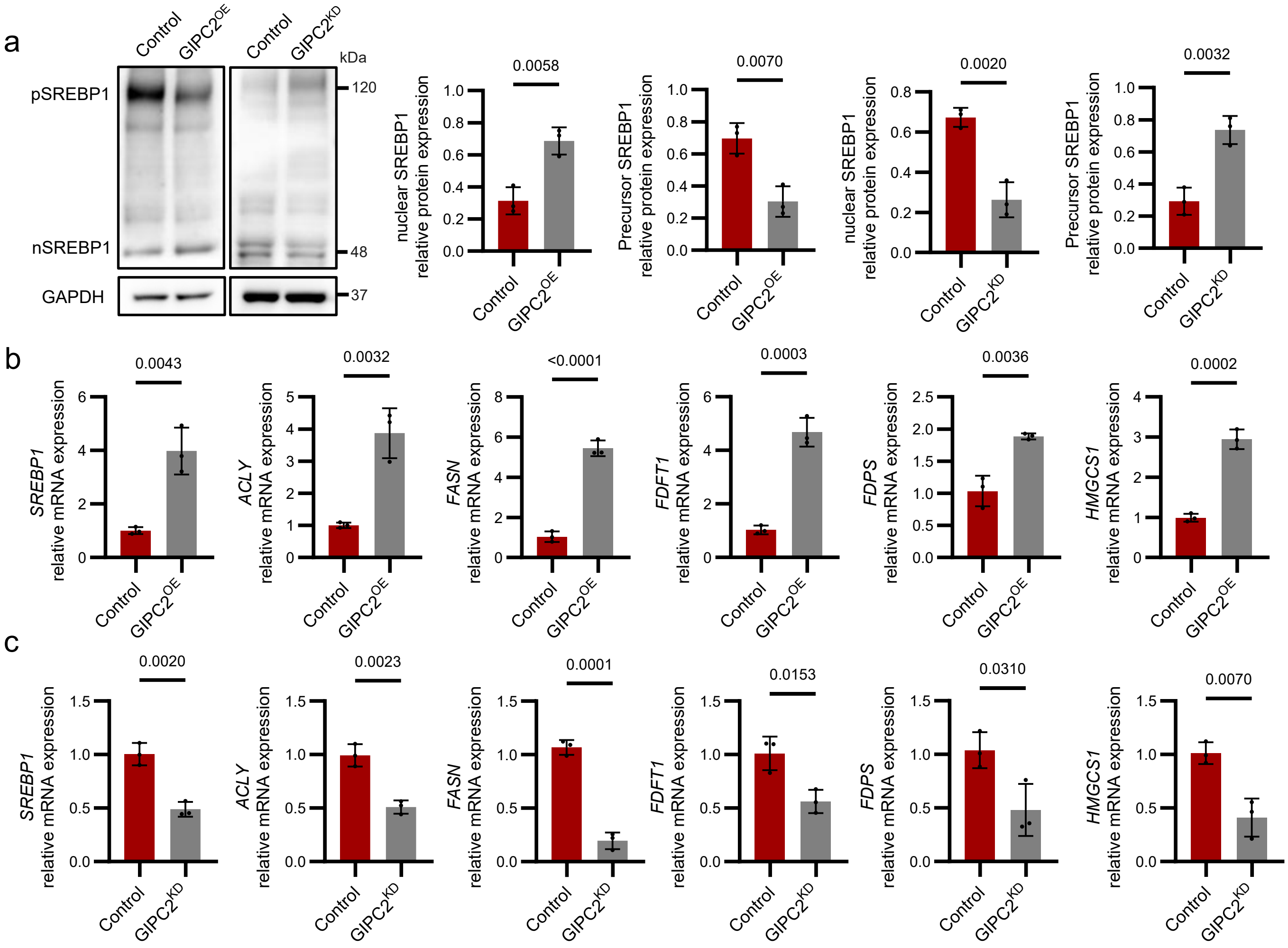

Supplementary Figure 14. GIPC2 promotes the synthesis of active nuclear SREBP1 (nSREBP1) and activates downstream target genes of SREBP1.

(a) WB analysis used to evaluate the protein levels of SREBP1 in GIPC2 overexpressing or knockdown cells. (b, c) qRT-PCR analysis of *SREBP1, ACLY, FASN, FDPS, FDFT1*, and *HMGCS1* in GIPC2- overexpressing or knockdown UC-MSCs. Statistical analysis: two-sided t test (a–c). Error bars: mean ± s.e.m.
