# Supplementary material for: GIPC2 regulation of the PKM2/SREBP1 signaling axis controls adipogenic differentiation of mesenchymal stem cells
Source: Cell Death Dis. 2026 Jan 7;17(1):5. doi: 10.1038/s41419-025-08088-9 (PMC12779644; doi:10.1038/s41419-025-08088-9)

Figure 1d

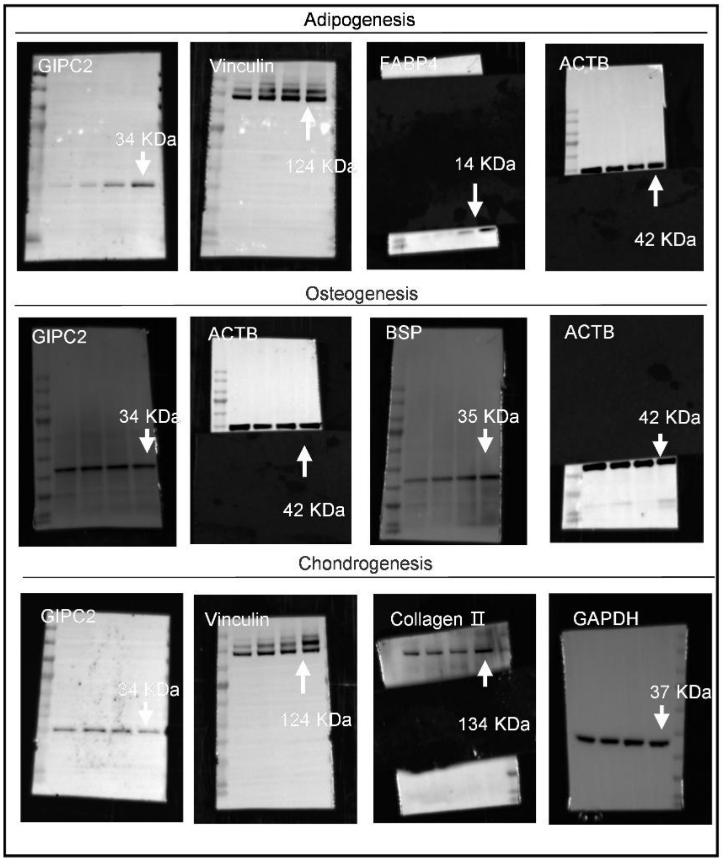

Figure 2c

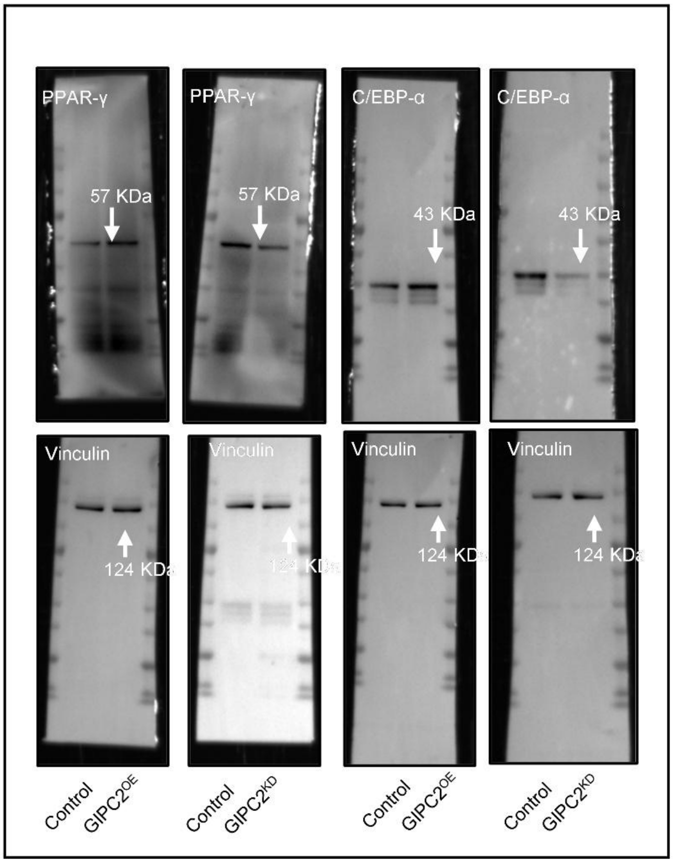

Figure 2c

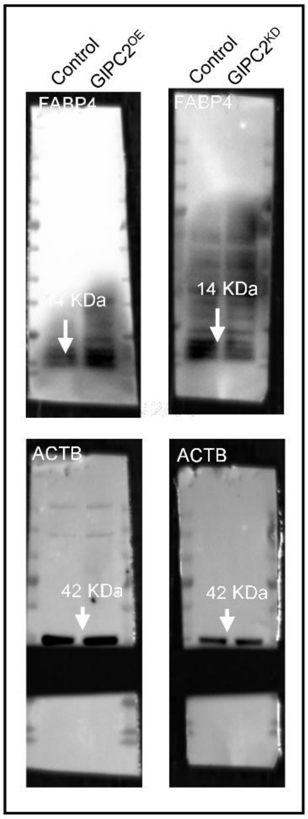

Figure 3c,f,g,h

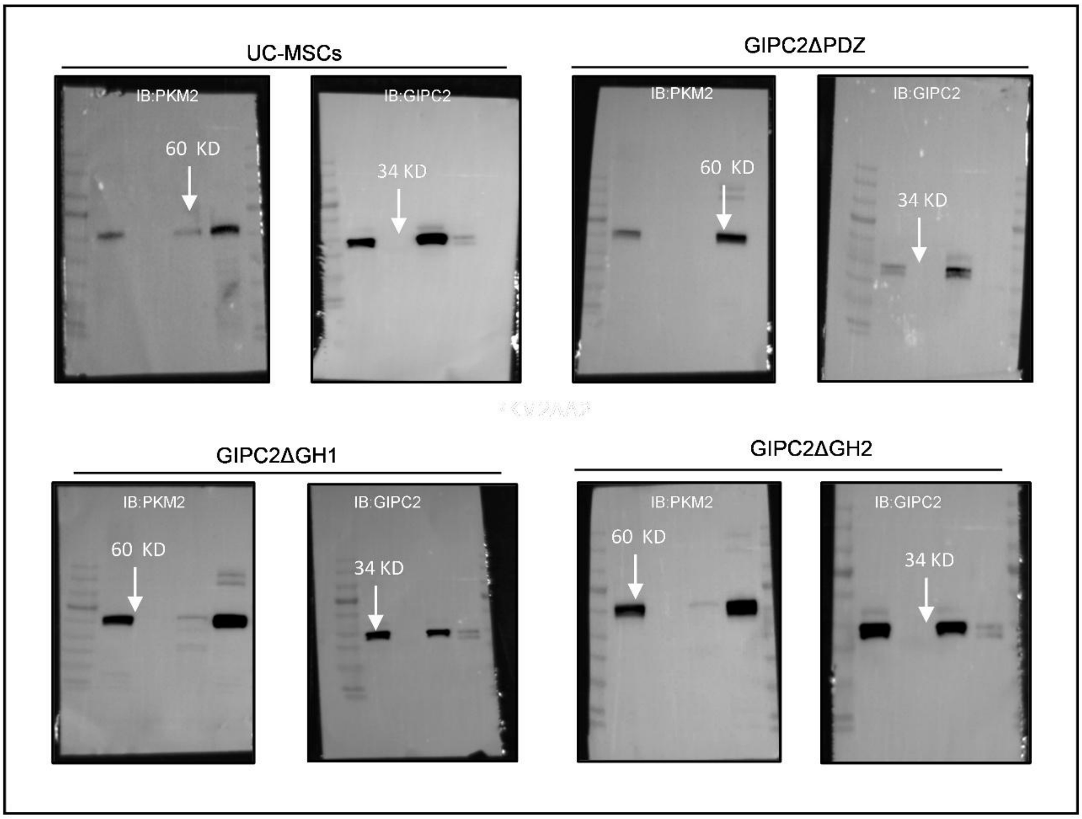

Figure 4b

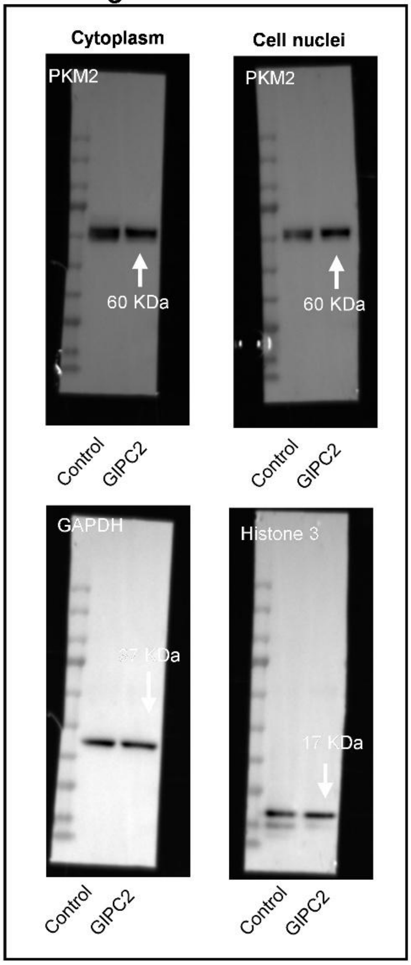

Figure 4c

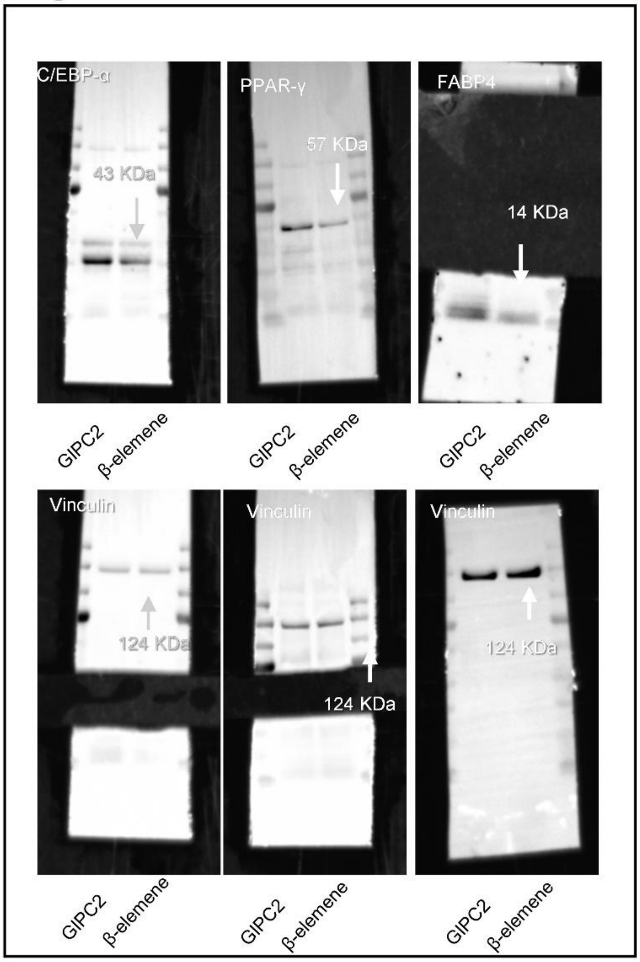

Figure 5d

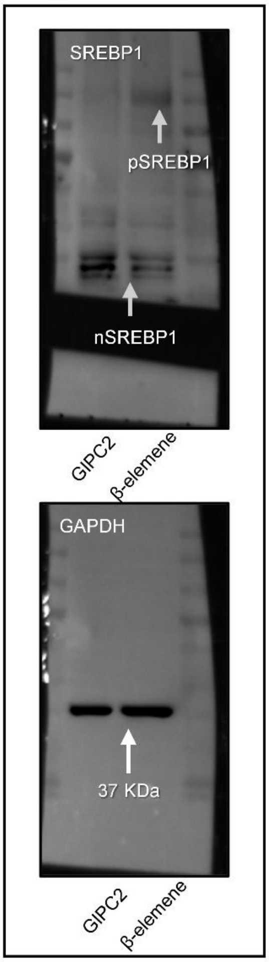

Figure 6a

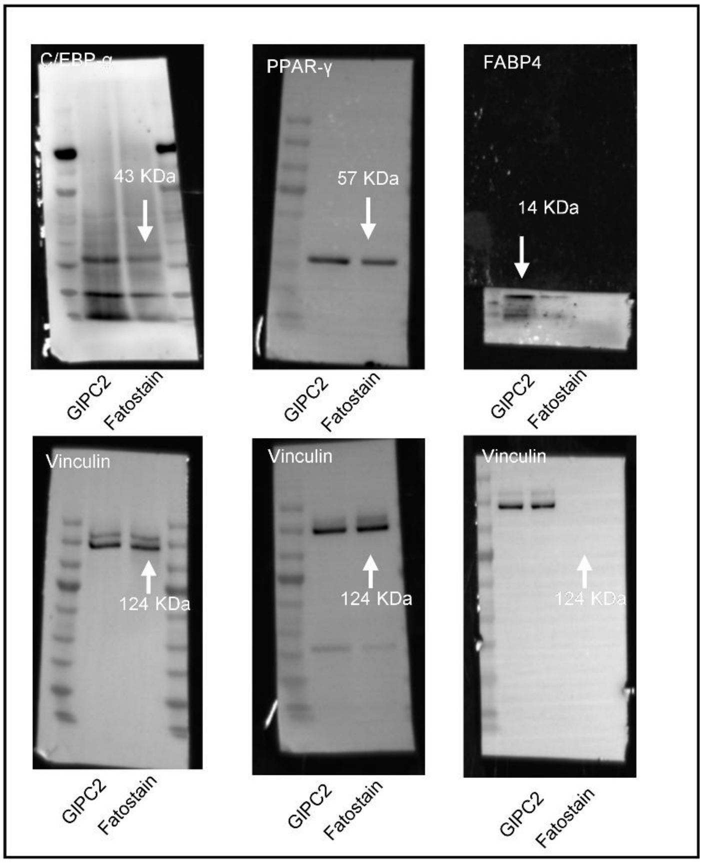

Figure 6c

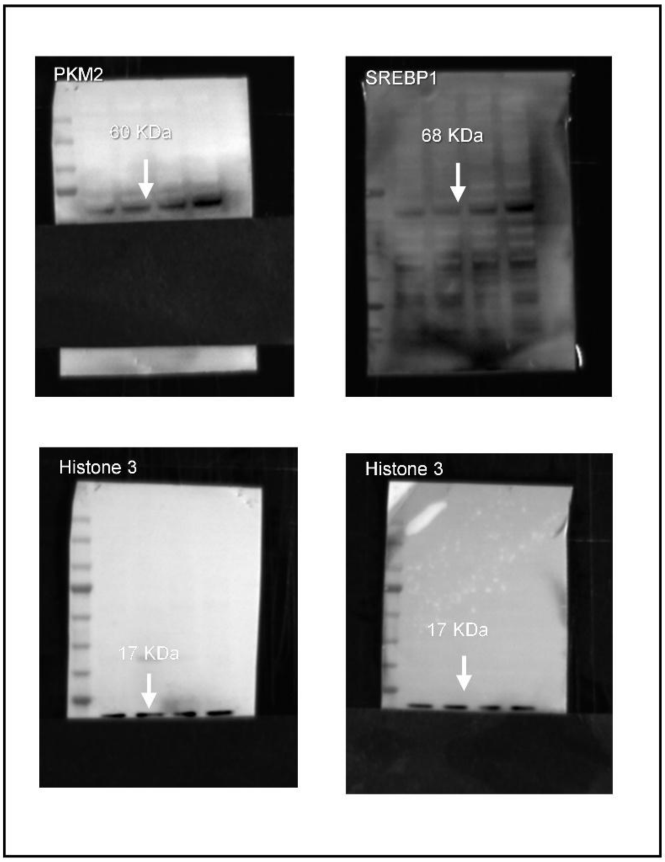

Supplementary Figure 1a

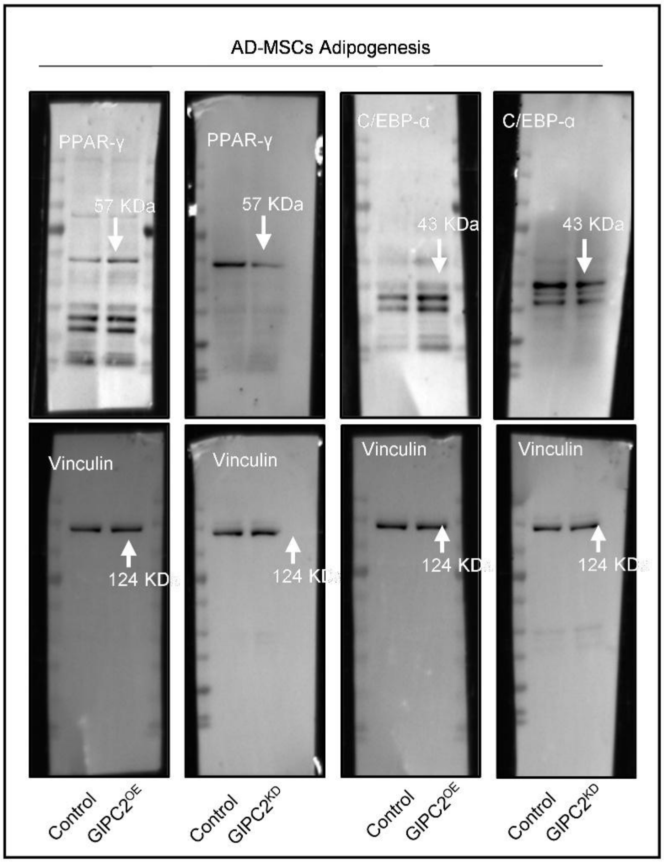

Supplementary Figure 1a

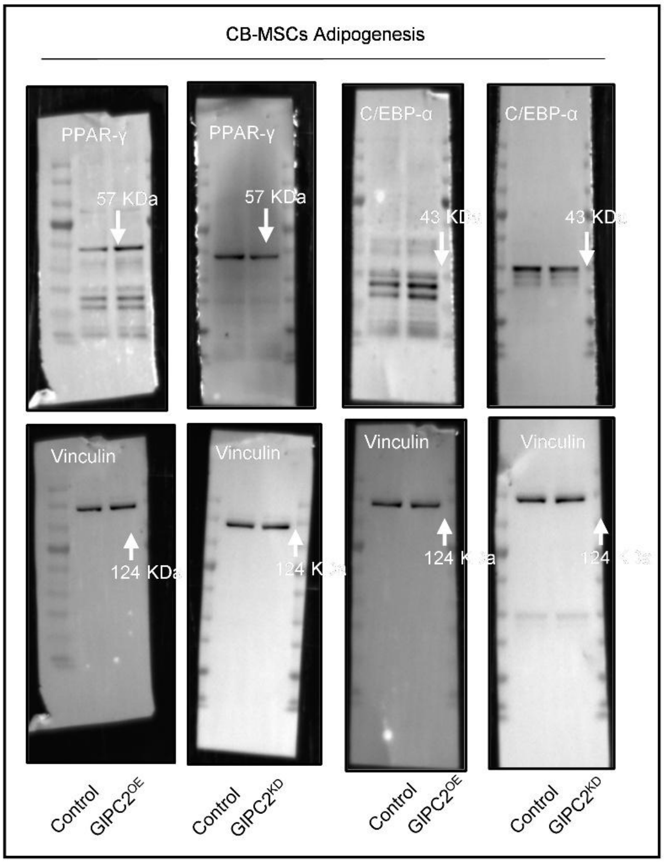

Supplementary Figure 1a

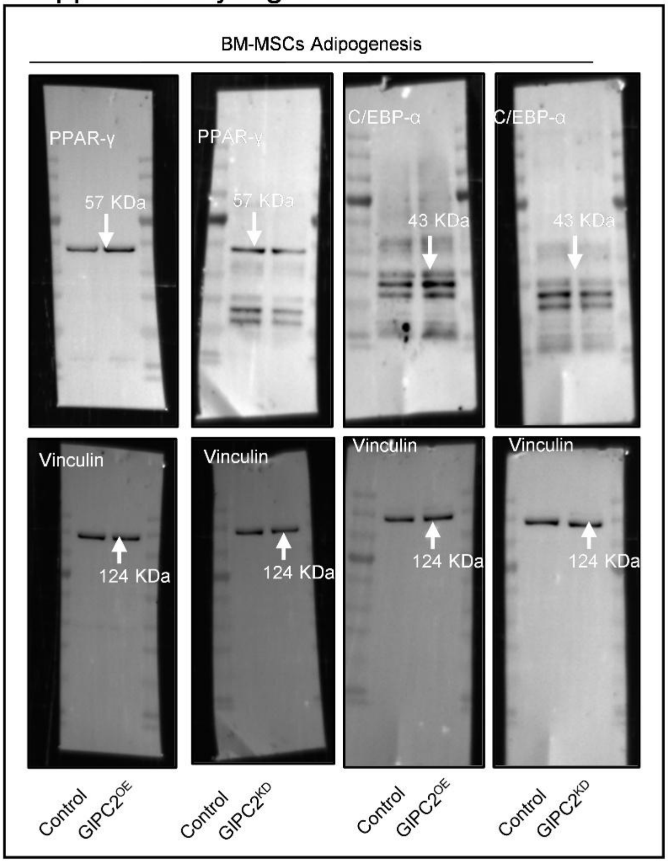

Supplementary Figure 1c

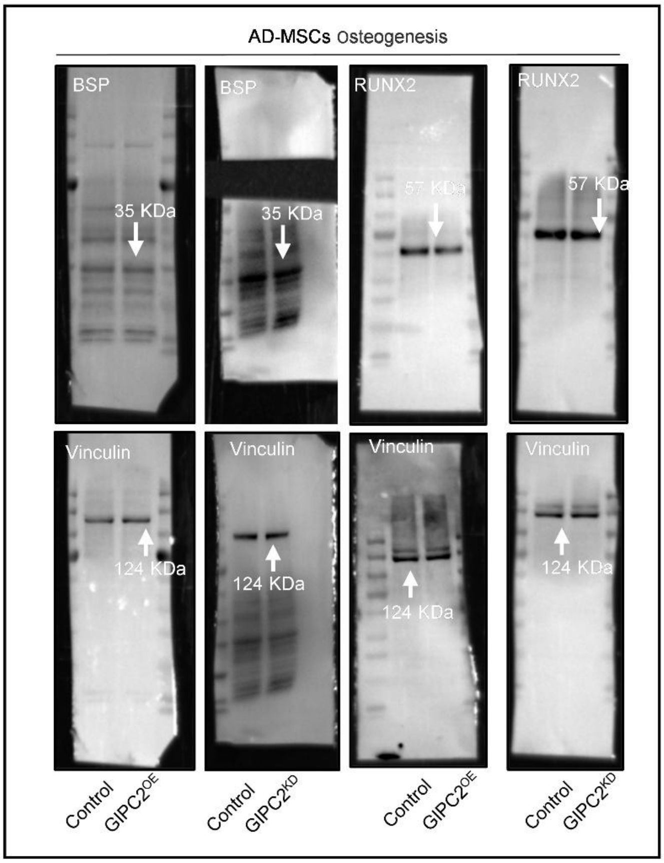

Supplementary Figure 1c

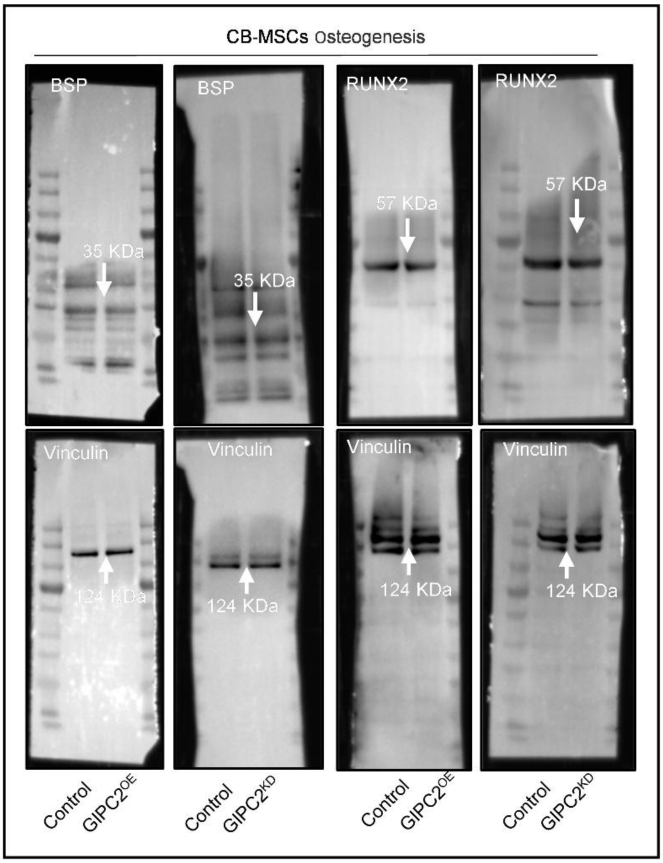

Supplementary Figure 1c

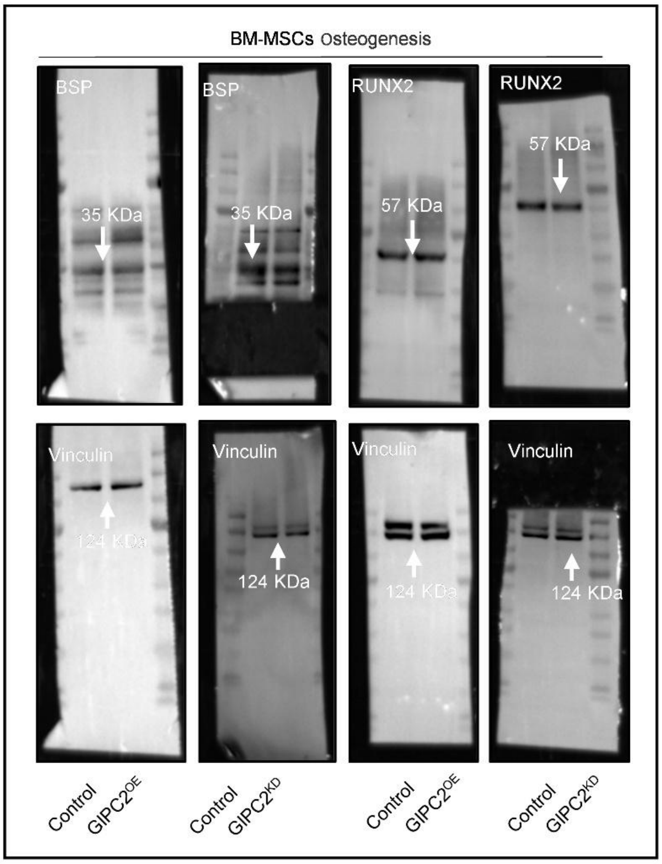

Supplementary Figure 1c

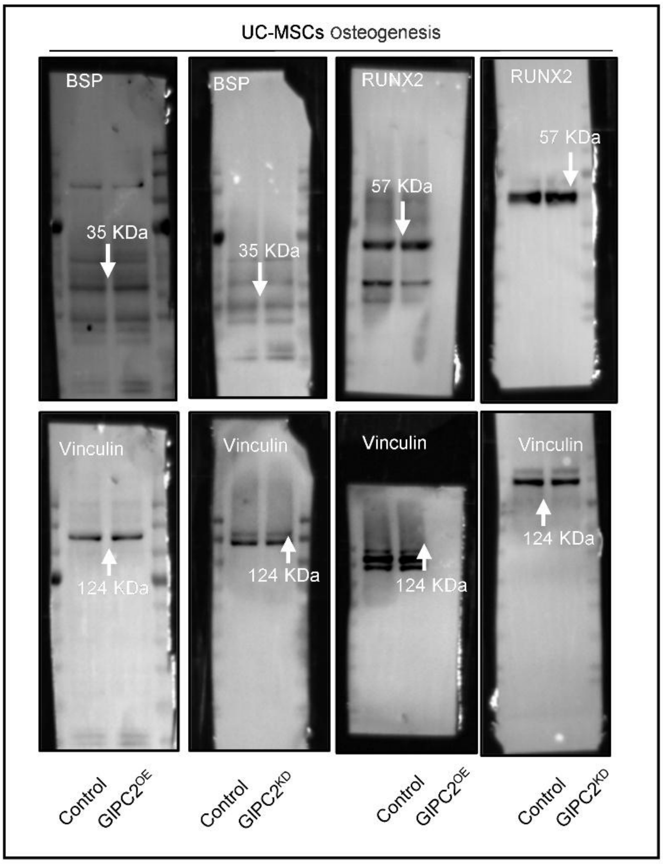

Supplementary Figure 2a

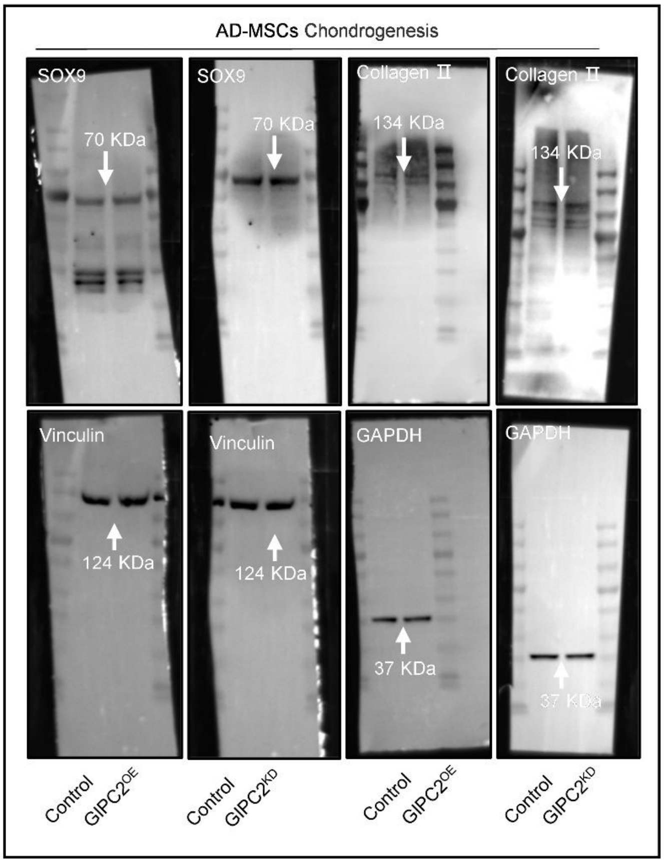

Supplementary Figure 2a

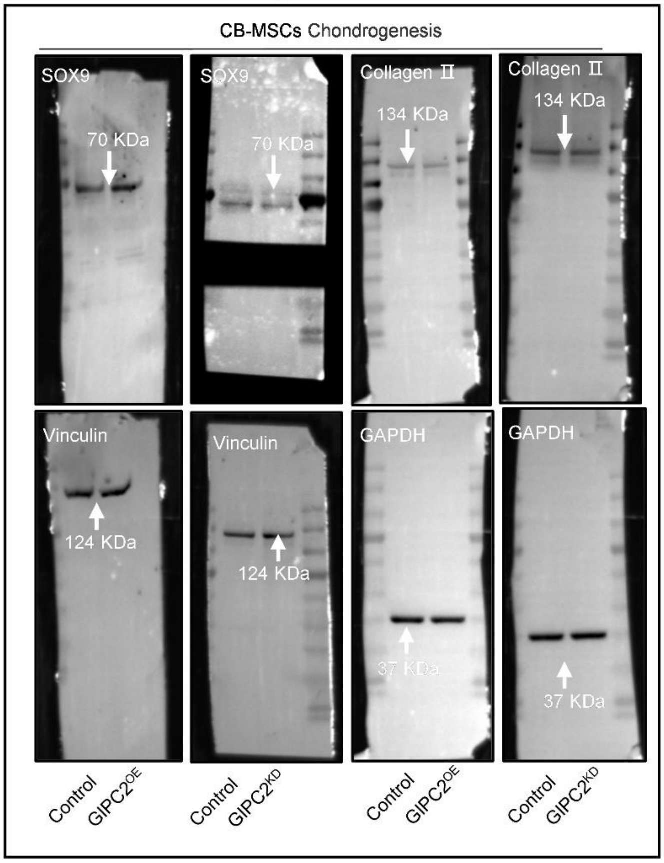

Supplementary Figure 2a

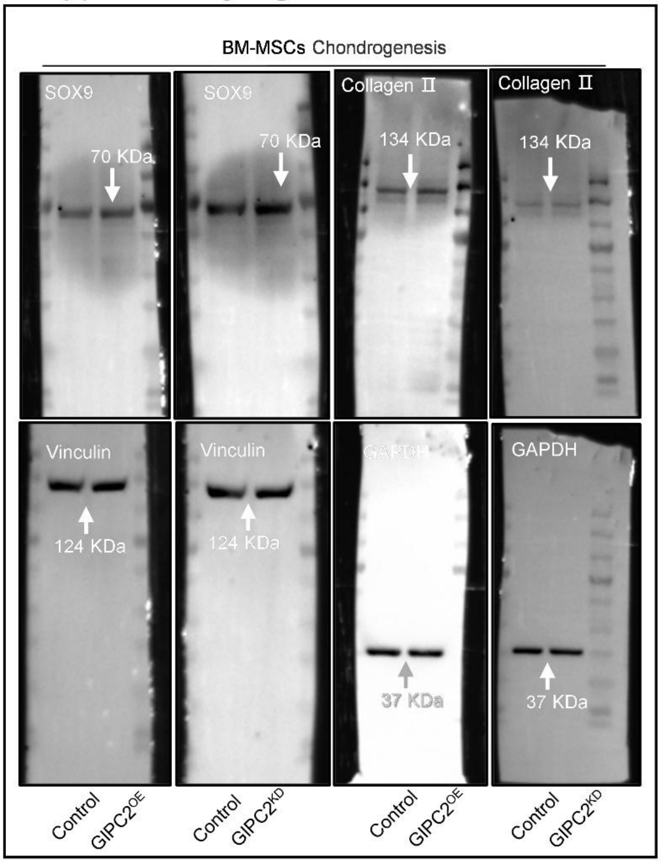

Supplementary Figure 2a

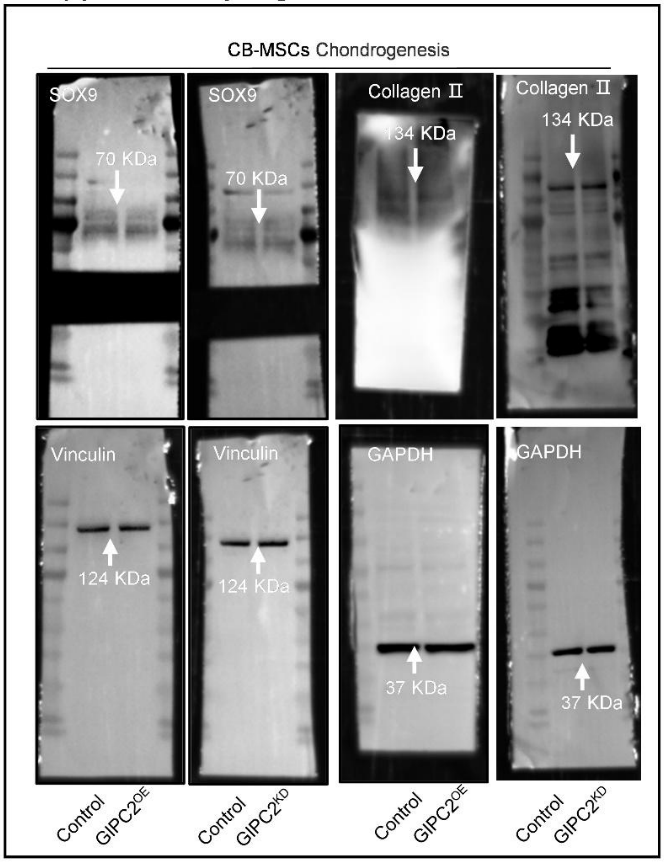

Supplementary Figure 5f

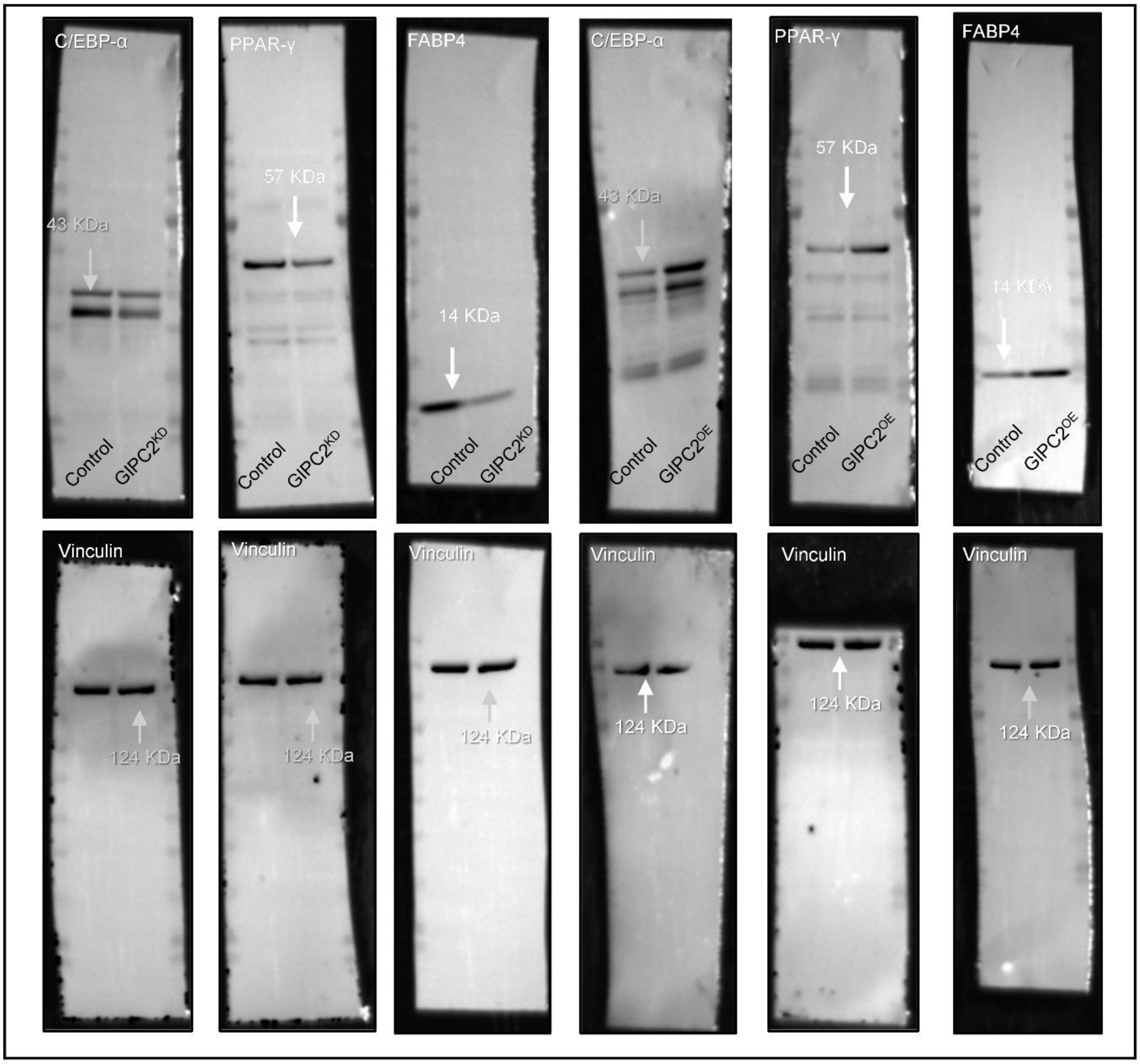

Supplementary Figure 7a

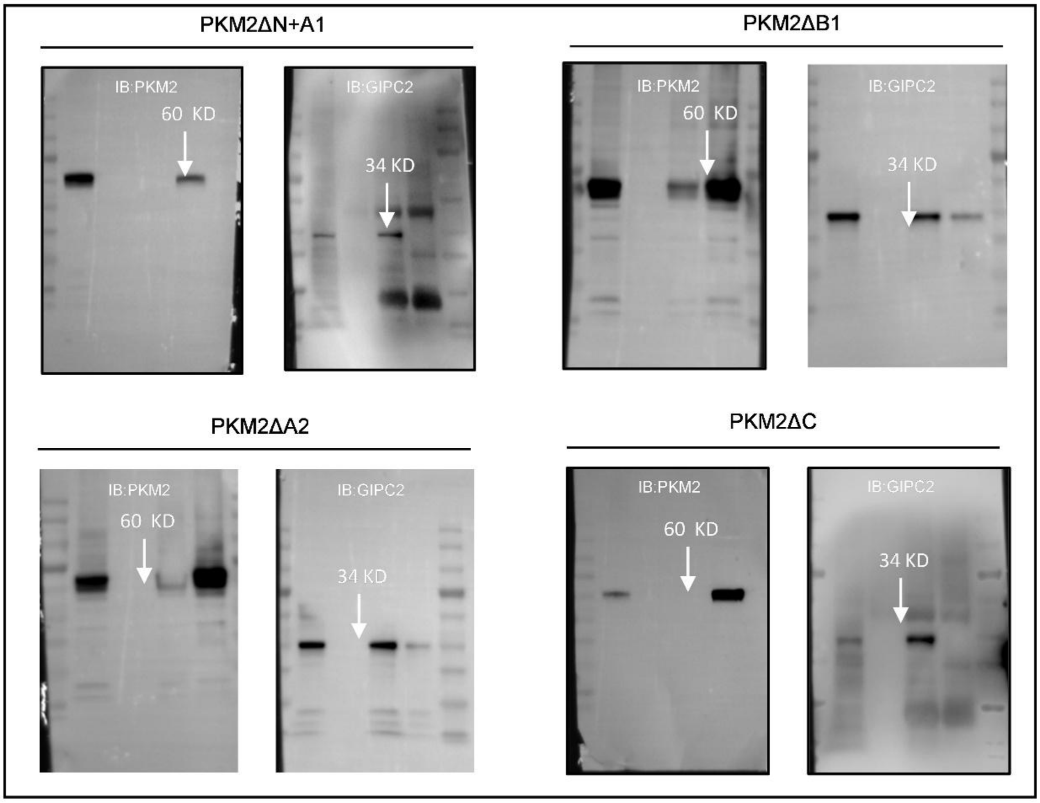

Supplementary Figure 8e-g

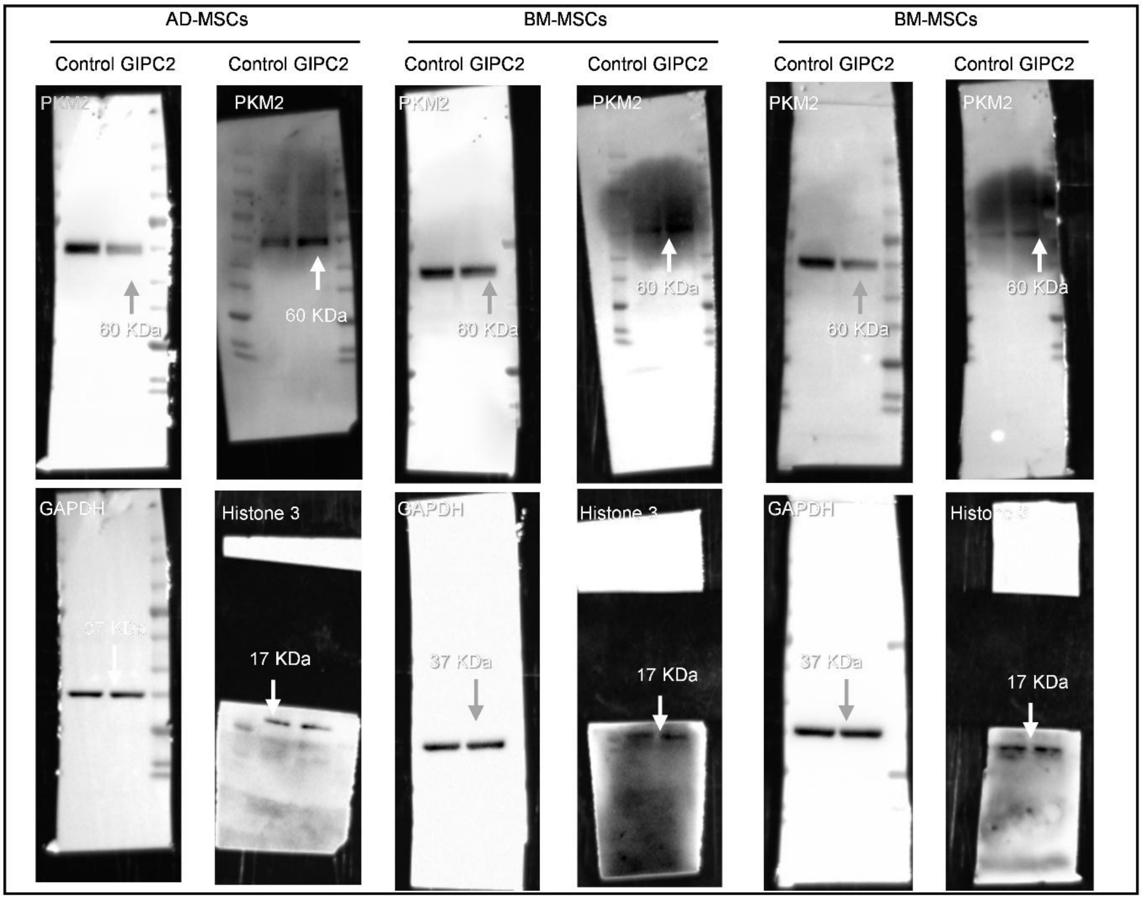

Supplementary Figure 9b,d

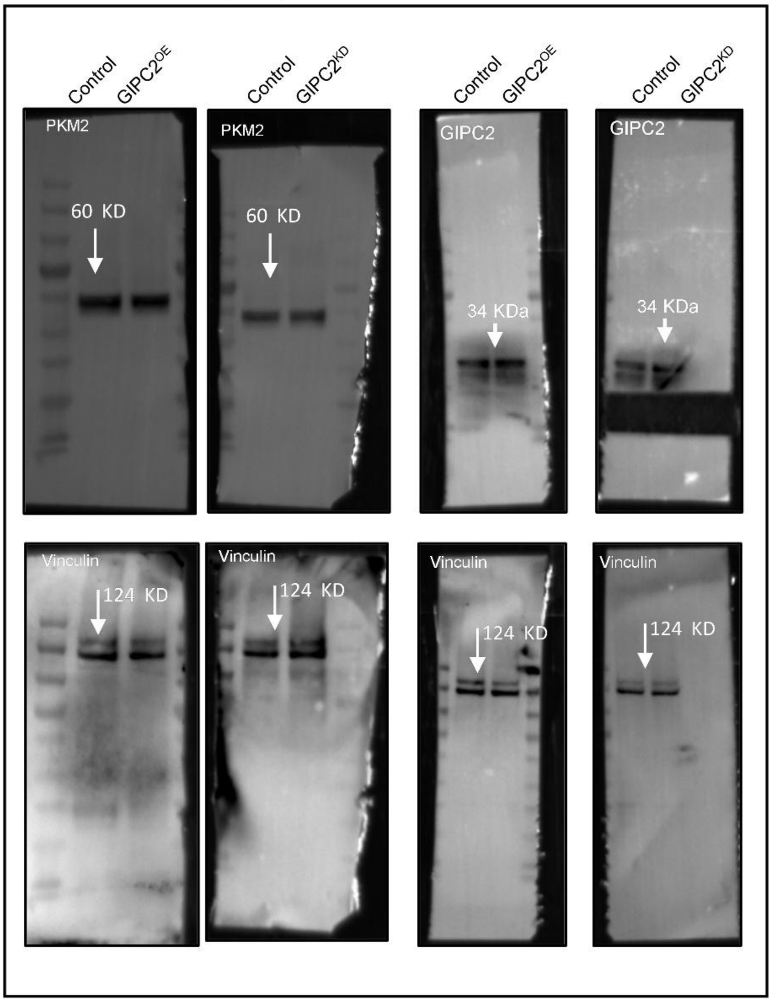

Supplementary Figure 11a

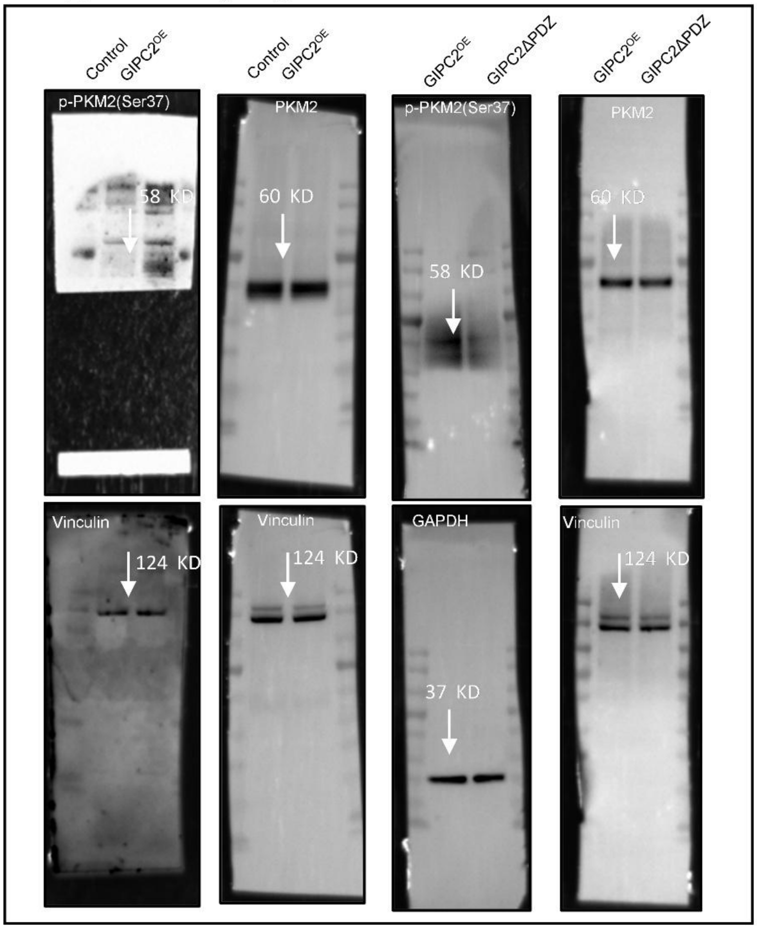

Supplementary Figure 11b,f

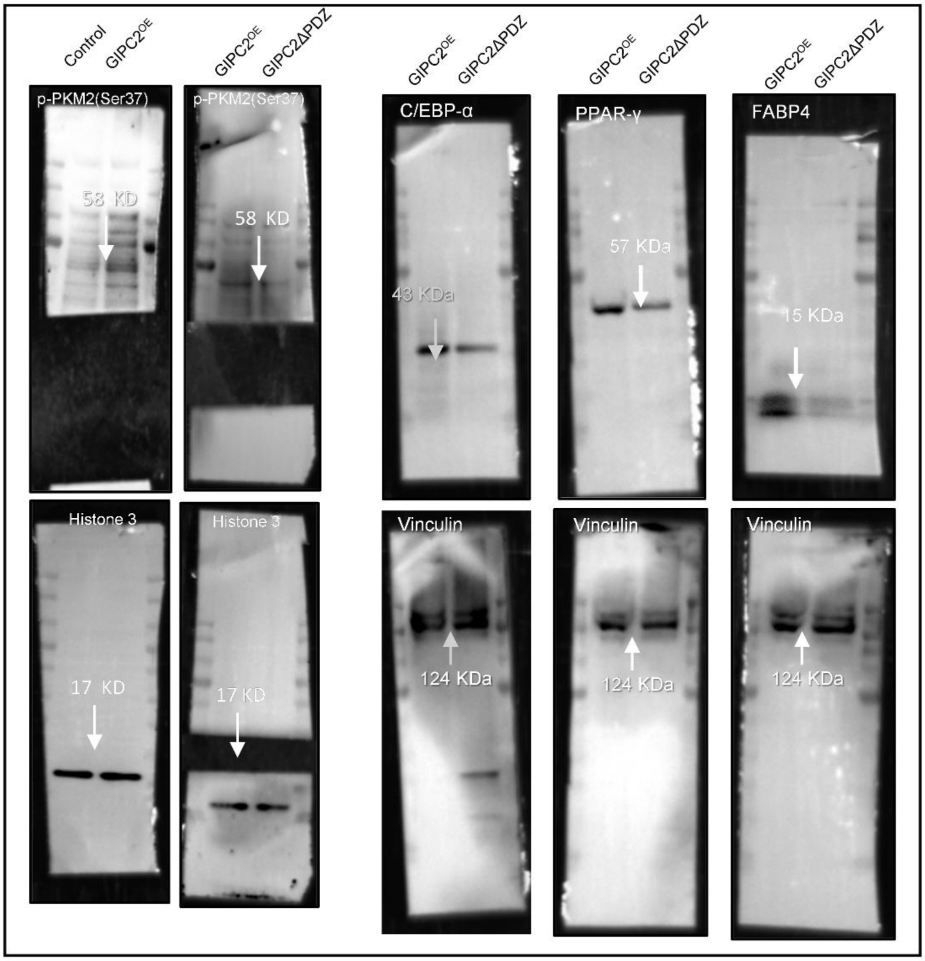

Supplementary Figure 13c,d

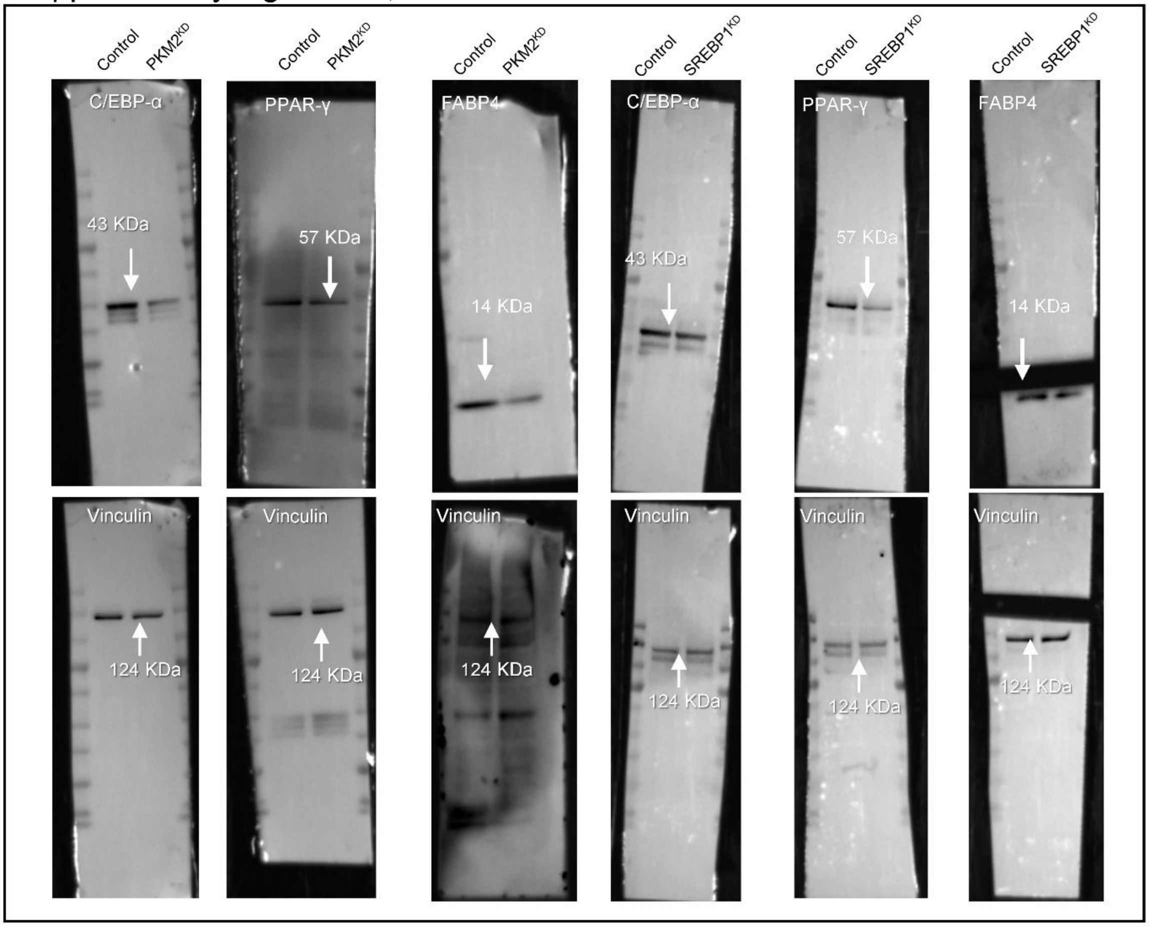

Supplementary Figure 13g-i

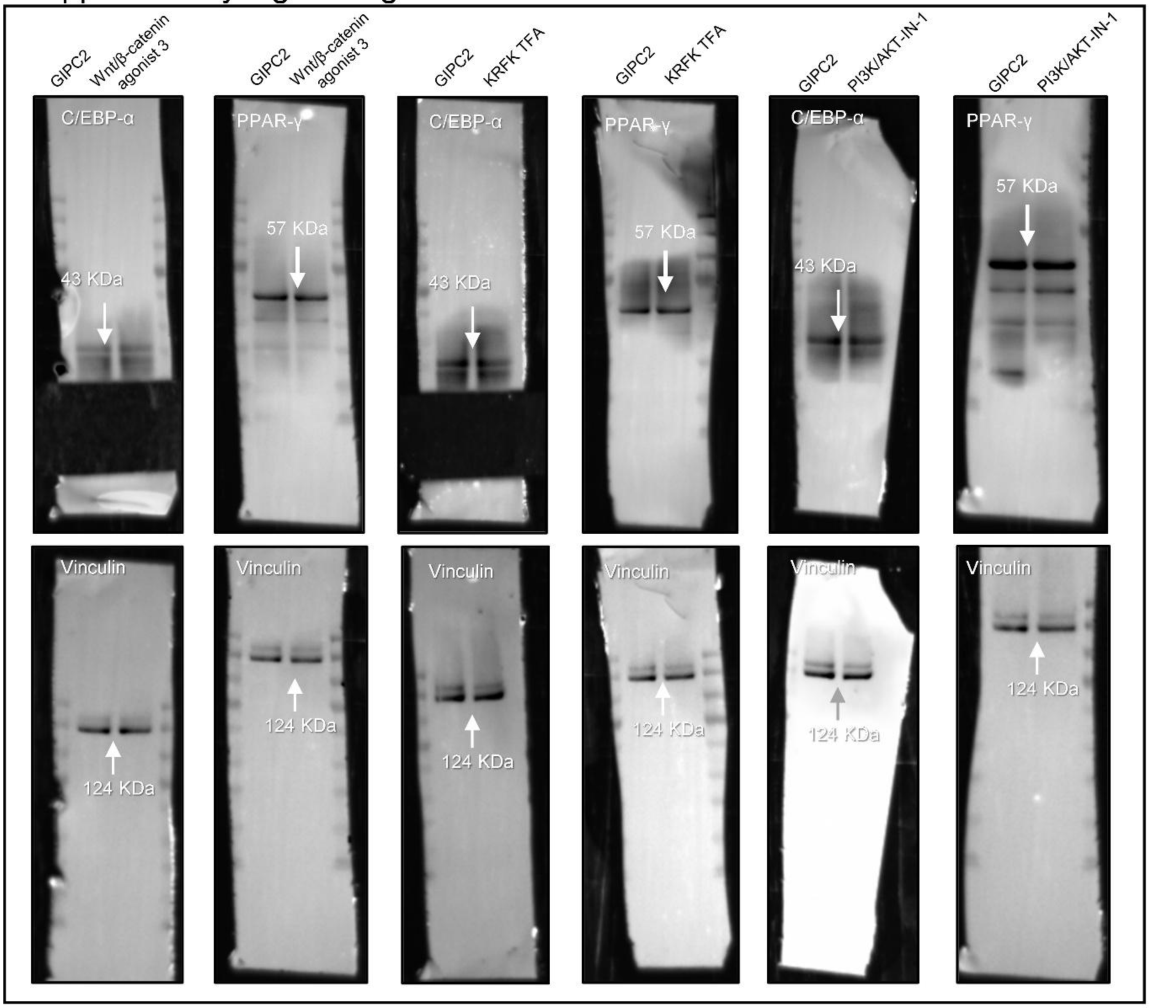

Supplementary Figure 14a

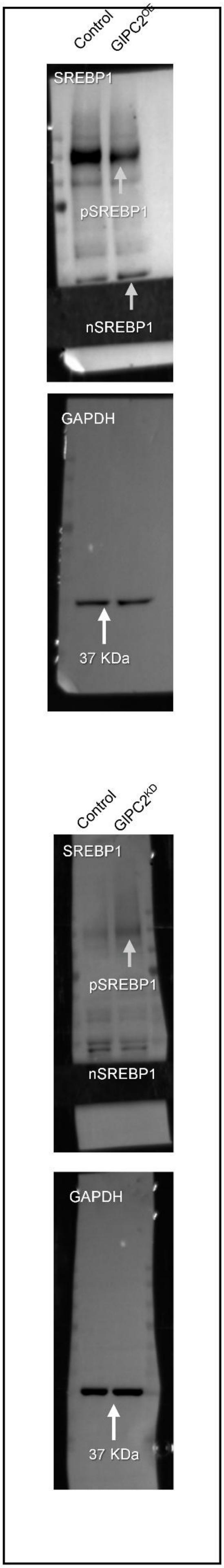

Supplement: Supplementary file 2 — original western blot [file 41419_2025_8088_MOESM2_ESM.pdf]
